# Cognitive behavioural therapy for the treatment of schizophrenia spectrum disorders: an umbrella review of meta-analyses of randomised controlled trials

**DOI:** 10.1016/j.eclinm.2023.102392

**Published:** 2024-01-05

**Authors:** Steven Berendsen, Silke Berendse, Jeanne van der Torren, Jentien Vermeulen, Lieuwe de Haan

**Affiliations:** a Department of Psychiatry, Amsterdam University Medical Center, Location Academic Medical Center, the Netherlands; b Dimence Mental Health Care, Deventer, the Netherlands; c Arkin Mental Health Care, Amsterdam, the Netherlands

**Keywords:** Schizophrenia, Psychosis, Cognitive behavioural therapy

## Abstract

**Background:**

Cognitive behavioural therapy (CBT) forms the standard psychotherapy for schizophrenia spectrum disorders (SSD). We aimed to summarize and evaluate the evidence on the effectiveness of CBT for SSD.

**Methods:**

In this umbrella review, we searched PubMed, Embase, Cochrane Database, and PsychInfo, for meta-analyses of randomised controlled trials (RCTs) of CBT in SSD published between database inception up to Aug 18, 2023. Inclusion criteria were RCTs investigating individually provided CBT in a population of patients with SSD, compared to either standard care, treatment as usually, or any other psychosocial therapies. No restrictions concerning follow-up or language were applied. We used the “assessment of multiple systematic reviews” (AMSTAR-2) appraisal checklist for the evaluation of methodological quality of meta-analysis. We extracted summary metrics from eligible studies in duplicate. The strength of evidence was classified by the sample size, p-value, excess significance bias, prediction intervals, significance of largest study, and heterogeneity. The strength of evidence was ranked according to established criteria as: convincing, highly suggestive, suggestive, weak, or not significant. Primary outcomes were general psychopathology, positive and negative symptoms. This study is registered in PROSPERO, CRD42022334671.

**Findings:**

We found 26 eligible meta-analyses, of which 16 meta-analyses provided sufficient data. Using the AMSTAR-2, we found limitations in details concerning the selection of study design, quality of the search and reporting of funding in included meta-analyses. A minority of 42.9% of the comparisons showed a significant result in favor of CBT; 57.1% were non-significant with no convincing or highly suggestive evidence. Suggestive evidence was found in favor of CBT for general psychopathology (6.2%, N = 34 RCTs, effect size (ES) = −0.33 (−0.47; −0.19), I^2^ = 67.93), delusions (16.7%, N = 27, ES = 0.36 (0.22; 0.51), I^2^ = 50.47), and hallucinations (33.3%, N = 28, ES = 0.32 (0.19; 0.46), I^2^ = 45.14) at the end of treatment (EoT). Weak (N = 34 RCTs, ES = −0.13 (−0.24; −0.02), I^2^ = 51.28), or non-significant evidence (N = 28 RCTs, ES = 0.12 (−0.03; 0.27) I^2^ = 64.63) was found for negative symptoms at EoT. At longer follow-up, evidence became weak or non-significant.

**Interpretation:**

Findings suggest that the effectiveness of CBT on general and positive symptoms in SSD at EoT was small to medium, while we found inconsistent evidence for a sustainable effect. CBT has no convincing impact on other relevant outcomes. Guidelines may use these results to specify their recommendations.

**Funding:**

None.

**Research in context**

**Evidence before this study**

Between January 2022 and August 2022 we searched PubMed and Cochrane Database of Systematic Reviews for umbrella reviews, systematic reviews and meta-analysis of randomised controlled trials (RCTs) concerning the effectiveness of cognitive behavioural therapy (CBT) vs any other control group for patients with schizophrenia spectrum disorders. Criteria for inclusion were individually provided CBT for people with schizophrenia spectrum disorders and investigating the effectiveness of CBT. We used the terms “psychosis,” “schizophrenia,” psychological treatment” with no language restrictions. Further, we applied the ‘assessment of multiple systematic reviews’ (AMSTAR-2) critical appraisal checklist for the evaluation of the methodological quality of meta-analysis. We identified a wide range of meta-analyses demonstrating conflicting evidence for CBT in the treatment of psychotic symptoms, relapse, and negative symptoms. Moreover, previous umbrella reviews examining psychological and psychosocial treatment in schizophrenia used a more global approach and did not stratify evidence according to regularly used classification models. Therefore, in the current study we applied a detailed umbrella approach on meta-analyses of RCTs specifically investigating CBT for clinically relevant outcomes in schizophrenia spectrum disorders.

**Added value of this study**

Based on a total of 26 meta-analyses of which 16 meta-analyses provided sufficient data to stratify the strength of evidence for specific outcomes. Suggestive evidence was found indicating that CBT has an effect on general psychopathology, delusions, hallucinations, and functioning at the end of treatment. At longer follow-up after treatment, evidence became inconsistent, weak, and non-significant findings were found. The majority of comparisons investigating negative symptoms, quality of life, or relapse were non-significant. By evaluating the methodological quality of the included meta-analysis, we found specific limitations in the selection of study design in the review, quality of the search and reporting of funding. Other important areas such as addressing heterogeneity, impact and discussion of bias, performing data-extraction in duplicate, and characterisation of the included population were done more frequently.

**Implications of all the available evidence**

Our results suggest that CBT has a small to medium effect on general and positive symptoms and functioning in schizophrenia spectrum disorders at the end of treatment, while evidence became inconsistent at follow-up. We found that CBT has no convincing effect on other clinically relevant outcomes. We recommend that clinical guidelines use the present findings to refine their endorsements. In addition, research should focus on methods to retain the effects of CBT on general and positive symptoms.

## Introduction

Cognitive behavioural therapy for psychosis (CBT) is widely recommended as individual psychotherapy for patients with schizophrenia spectrum disorders (SSD). The National Institute for Health and Care Excellence (NICE),^1^ the American Psychiatric Association,^2^ and the Dutch clinical guideline for psychosis^3^ advise CBT as routine psychotherapy for the treatment of positive symptoms, negative symptoms, and improvement of other clinically relevant outcomes. Despite strong advocacy for inclusion of CBT in guidelines, its scientific basis concerning effectiveness remains controversial.^4^ Several meta-analyses attempted to provide more definitive answers on the effectiveness of CBT for schizophrenia and related psychosis. Results range from null-findings^5^ to small,^6^–and moderate effect sizes^7^^,^^8^ in favor of CBT against treatment as usual, mixed, or active conditions. The reported discrepancies between meta-analytic findings were explained by differences in included populations, variation in severity of psychotic symptoms, methodological study quality, or primary study outcome.^9^^,^^10^

Conflicting meta-analyses concerning medical conditions are widespread and may lead to either under or–overtreatment. CBT for SSD is already included in clinical guidelines. Although implementation of guidelines is incomplete, a risk for over-treatment is present and possibly accompanied by unnecessary loss of financial and clinical resources. Umbrella reviews offer an opportunity to resolve conflicting findings, by providing a summary overview of current evidence, investigating methodological quality of meta-analysis, within study-bias, and generating a hierarchy of evidence. This may inform and support clinicians and developers of clinical guidelines on the current state of evidence for specific treatments. A detailed umbrella analysis on CBT in the treatment of schizophrenia spectrum disorders is currently missing.

In the present study we applied the umbrella technique to available meta-analyses of randomised controlled trials (RCTs) investigating the effect of CBT vs any control conditions for SSD. The aim is to create an overview of all existing evidence in an area of inconsistent findings. Secondly, we set out to examine results from meta-analyses in terms of significance-level, sample size and study parameters to create a hierarchy in strength of evidence.

## Methods

### Search strategy and selection criteria

The clinical librarian was consulted before conducting the search for the umbrella review. The following databases were searched for eligible meta-analysis: MEDLINE, Cochrane, Embase, PsychINFO from inception to 18 august, 2023. The search strategy used the terms “schizophrenia spectrum disorders”, “psychosis”, “cognitive behavioral therapy,” and “meta-analysis”. If suitable for the umbrella review, we adhered to the PRISMA guidelines for systematic reviews. A detailed description of the search script is provided in the supplemental list 1. Two authors (SBe, JMT) independently screened titles, abstract, full text articles, and disagreements were resolved by discussion with a third researcher (SBn). Meta-analysis of RCTs investigating the effect of individually provided CBT in a population of patients with at least 70% SSD, compared to either standard care/treatment as usually/waiting list, or any other psychosocial therapies were included. Primary outcomes were general psychopathology, positive and negative symptoms, delusions, and hallucinations. Secondary outcomes were affective symptoms, anxiety, depression, social functioning, functioning and distress, relapse, rehospitalisation and quality of life, no restrictions concerning follow-up were applied. No language restrictions were applied. The minimum number of RCTs in the meta-analysis in order to be included was three. We decided to use this criterion to be able to include meta-analyses concerning less frequent evaluated but clinically relevant outcome measures. To evaluate whether this criterion substantially influenced results we performed a sensitivity analysis in which we restricted the analysis for each outcome to meta-analysis that only include >20 RCTs. Study patients had to be of adult age. Cross-over or quasi-randomised RCTs, observational, cohort, or case–control studies were excluded. All meta-analyses fulfilling the criteria, along with overlapping studies, were included.

The protocol of the study was registered in PROSPERO (no. CRD42022334671) on 25th of May 2022.

### Data analysis

Two authors (SBe, JMT) independently performed the data extraction and disagreements were resolved by consulting a third author (SBn). From each meta-analysis, the following information was extracted: first author, year of publication, DSM classifications, number of included RCTs, total sample size, type of intervention, definition of control condition, length of follow-up, summary effect size with confidence intervals (i.e., standardized mean difference, relative risk, odds ratio, hazard ratio). Subsequently, the individual effect sizes of each RCT with its 95% confidence interval, standard error and sample size were extracted. We did not contact individual authors for additional data, only for clarification concerning follow-up when it was not provided in the published report. This data was used for ranking the evidence and assessment of within-study parameters. We did not combine any data from meta-analyses. The ‘assessment of multiple systematic reviews’ (AMSTAR-2) critical appraisal checklist for evaluating the methodological rigor of meta-analysis was used.^11^ The AMSTAR-2 assesses quality by scoring (i.e., yes, no, or partial yes) 16 items related to bias assessment or quality of the search of the meta-analysis. We did not apply the proposed classification (‘high’, ‘moderate’, ‘low’, or ‘critically low’ quality) of the AMSTAR-2 nor the total score of the 16 items. This system leads to a quick downgrading of meta-analysis to critically low while other methodological aspects may actually be good. Instead, we will we describe the percentage of meta-analyses that scored positive on an item of the AMSTAR-2, to provide an overall picture of several methodological aspects of the included meta-analyses. Detailed information concerning criteria for each individual item can be found the publication by Shea et al., 2017.^11^ Two authors (SBe and JMT) independently applied the AMSTAR-2 on each eligible meta-analysis. The scores were discussed with a third author (SBn) and disagreements were resolved.

The browser-based statistical program (metaumbrella.org) developed by Goslin, Solanes Font, Fusar-Poli and Radua was used, following guidelines for umbrella reviews.^12^^,^^13^ This application is specifically designed to perform umbrella reviews (R-scripts available online).^14^ Extracted data from individual RCTs provided by each meta-analysis were entered in the browser-based program. To perform statistical analysis data concerning sample size, effect size plus confidence interval, or standard error was necessary. No statistical analysis was performed if these metrics were not provided in the published meta-analysis.

In the first step, random-effects meta-analysis (restricted likelihood maximum^15^) were repeated to transform the reported effect sizes in one common effect size (Hedges’ G) with 95% confidence interval.

In the second step, a number of study parameters were assessed: 1) heterogeneity was calculated with I^2^ (low heterogeneity less than 50%, high heterogeneity more than 50%). The existence of large heterogeneity could suggest that there are two or more distinct groups investigating patients, and the results of a meta-analysis would not accurately represent either of the groups. 2) Egger's small-study effect was used to evaluate whether small studies had larger effects sizes compared to larger studies. 3) Excess significance bias per summary estimate was calculated by evaluating if the observed studies with significant results were different from the expected number of studies with significant results. 4) The prediction interval was calculated by estimating the range of the effect size of future studies. The prediction interval is also a form of heterogeneity. The clinical interpretation of for example τ^2^ remains difficult as high heterogeneity does not necessarily mean high variation in study effects. The prediction interval also presents heterogeneity but indicates the uncertainty for the effect that would be expected in a new study examining the same association. Another advantage of the prediction interval is that it is expressed in the same metric as the original effect size measure. 5) We determined whether the largest study included in the meta-analysis was significant, assuming that the most plausible effect was given by the largest study. 6) For each non-significant finding, an estimation was made to ascertain whether the comparison was sufficiently powered (>80%) for three effect sizes (0.3, 0.5, 0.8). Two authors (SBe, JMT) independently performed all analysis.

In the third step, evidence was stratified by the commonly applied classification model used in previous umbrella reviews.^16^, ^17^, ^18^, ^19^ Classes were determined by the sample size, p-value, and study parameters. Subsequently, we will calculate the percentage of comparisons that fall in each class of evidence. For example: in total 50 comparisons were made between CBT and TAU with outcome delusions. 10 out of 50 comparisons were significant and fit in class of evidence IV (weak evidence), indicating that 10/50 = 20% of the comparisons with regard to delusions showed weak evidence.

Class I convincing evidence: >1000 cases receiving CBT, p-value < 1 × 10^−6^, no small-study effects, no excess significance bias, prediction intervals not including null, largest study significant, I^2^ < 50%. Class II highly suggestive evidence: >1000 cases receiving CBT, p-value < 1 × 10^−6^, largest study significant. Class III suggestive evidence: >1000 cases receiving CBT, p-value 1 × 10^−3^, and class I–II criteria not met; Class IV weak evidence: all comparisons p-value < 0.05. Non-significant p-value ≥ 0.05.

In the final step we will conduct a sensitivity analysis. Here, we restrict the analysis to comparisons between CBT and any other control group that consist of more than 20 RCTs and compare these to our primary findings.

### Role of the funding source

There was no funding source for this study. The corresponding author had the final responsibility for the decision to submit for publication.

## Results

**Fig. 1:**
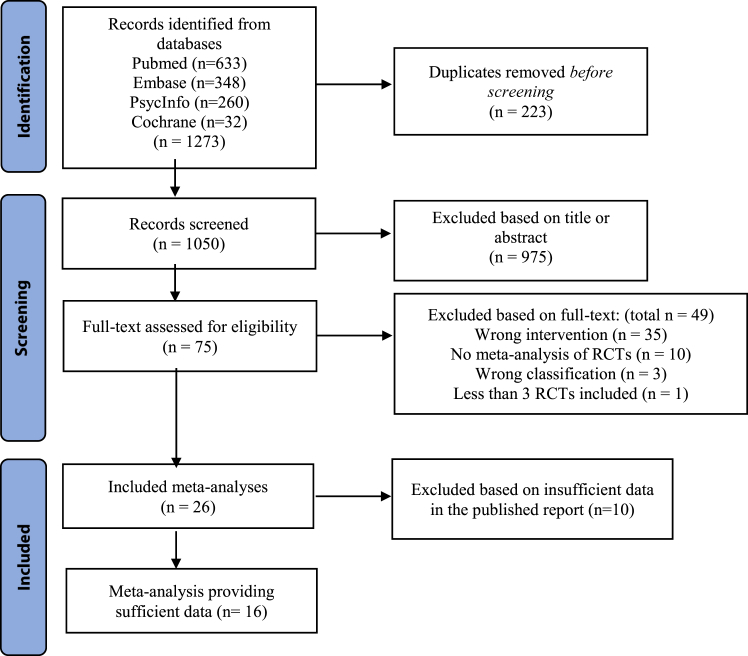
Flow Diagram for study screening and selection.

The search yielded a total of 1273 hits, of which 1050 publications were screened by title and abstract after eliminating duplicates. Subsequently, 75 full-text publications were read in full-text, resulting in inclusion of 26 meta-analyses of RCTs. Fig. 1 depicts a flow diagram demonstrated the search process and details on exclusion of studies. The total list of included meta-analyses and search details were provided in the supplemental list 1 and 2. 16 of the 26 meta-analyses provided sufficient data to perform the appropriate umbrella analysis, the list of the latter meta-analyses is provided in the supplemental list 3. General characteristics of the selected meta-analyses are shown in Table 1. Reasons for exclusions of full-text publications are provided in Supplemental Table S1. From the 16 meta-analyses, we extracted a total of 70 comparisons of CBT vs any other control group. Findings concerning CBT vs any control group described in the remaining 10 meta-analyses that did not provide sufficient data (sample size, effect size plus confidence interval, or standard error) to perform analysis were reported in Supplemental Tables S2 and S3. We could not analyse these findings, because insufficient data were available in these reports. The sensitivity analysis is shown in Supplemental Table S4. The number of times a RCT was included in the umbrella analysis is shown in Supplemental Table S5.

**Table 1:** General characteristics of selected meta-analyses.

	Number of databases searched	Outcomes	Number of primary studies	Sample size (total)	Country
Barnicot et al., 2020	3	General psychopathology; Positive symptoms; Social functioning	7	Unknown	England: United States
Bighelli et al., 2018	8	General psychopathology; Positive symptoms; Negative symptoms; Depressive symptoms; Functioning and distress; Quality of life	53 (total)	4068 (total)	Germany; Switzerland; Italy; Japan
Bighelli et al., 2021	7	General psychopathology; Positive symptoms; Negative symptoms; Depressive symptoms; Relapse/rehospitalisation; Functioning and distress; Suicide	72 (total)	10,364 (total)	Germany; Italy; Spain; Switzerland; Japan
Bighelli et al., 2023	8	Functioning	58	5048	Germany
Burns et al., 2014	4	General psychopathology; Positive symptoms	12	639	Canada
Jauhar et al., 2014	4	General psychopathology; Positive symptoms; Negative symptoms; Hallucinations	52	Unknown	Enland; Spain; Canada
Jones, Hacker, Meaden et al., 2018	7	General psychopathology; Positive symptoms; Negative symptoms; Affective symptoms; Rehospitalisation; Relapse; Functioning and distress	36	3542	England China
Jones, Hacker, Xia et al., 2018	7	General psychopathology; Positive symptoms; Negative symptoms; Anxiety; Rehospitalisation; Relapse; Functioning and distress	60	5992	England; China
Kennedy et al., 2017	3	Hallucinations	2	105	England
Laws et al., 2018	2	Functioning and distress; Quality of life	37	1579	England; Spain
Lincoln et al., 2008	2	General psychopathology; Positive symptoms; Negative symptoms; Depressive symptoms; Rehospitalisation; Functioning and distress	18	1667	Germany
Lutgens et al., 2017	5	Negative symptoms	26	Unkown	Canada
Lynch et al., 2010	3	General psychopathology; Relapse	9	581	England; Spain
Mc Glanaghy et al., 2021	4	General psychopathology	25	1477	England; The Netherlands
Mehl et al., 2015	5	Delusions	19	Unknown	Germany
Naeem et al., 2016	8	General psychopathology; Delusions; Hallucinations; Quality of life	9	1207	Canada; England
Newton-Howes et al., 2013	3	General psychopathology	9	602	England; New Zealand
Pilling et al., 2002	9	General psychopathology; Relapse/rehospitalisation	8	393	England
Sarin et al., 2011	3	General psychopathology; Positive symptoms; Negative symptoms; Hallucinations; Depressive symptoms	22	2469	Sweden
Todorovic et al., 2020	5	General psychopathology Positive symptoms Negative symptoms	4	525	Australia
Turner, Reijnders et al., 2020	4	Negative symptoms	14	898	The Netherlands; England; Germany; Canada; Spain; Brazil; United States; Australia; China
Turner, Burger et al., 2020	4	Delusions Hallucinations	35	2407	The Netherlands England
Turner, van der Gaag et al., 2014	4	General psychopathology; Positive symptoms; Negative symptoms	22	706	The Netherlands
van der Gaag et al., 2014	3	Delusions; Hallucinations	18	1418	The Netherlands; England
Velthorst et al., 2015	3	Negative symptoms	30	2312	The Netherlands
Wykes et al., 2008	6	Positive symptoms; Negative symptoms; Functioning and distress	34	1964	England

### Methodological quality

AMSTAR-2 scores of 26 meta-analyses are shown in Table 2. We will describe the percentage of meta-analysis that have a partial yes or full yes on each item of the AMSTAR-2. All meta-analyses described the Problem; Intervention; Comparison; Outcome (PICO), 7 meta-analyses (27%) had a pre-registration protocol, 4 meta-analyses (15%) explained the selection of study design for inclusion, 16 meta-analyses (62%) used a comprehensive literature search, 14 meta-analyses (54%) did the study selection in duplicate and 18 meta-analyses (69%) did the data extraction in duplicate. 8 meta-analyses (31%) provided a list of excluded studies and justified the exclusions, 22 meta-analyses (85%) described the included population in adequate detail, 19 meta-analyses (73%) used a satisfactory technique for assessing the risk of bias, 2 meta-analyses (8%) reported on the sources of funding for the studies included in the review. 18 meta-analyses (69%) used appropriate methods for statistical combination of results, 18 meta-analyses (69%) assessed the potential impact of risk of bias in individual studies on the results of the meta-analysis, 17 meta-analyses (65%) accounted for risk of bias in individual studies when discussing the results, 19 meta-analyses (73%) provided a satisfactory explanation and discussion of any found heterogeneity, 18 meta-analyses (69%) carried out an adequate investigation of publication bias, and 20 meta-analyses (77%) reported any potential sources of conflict of interest, including any funding received for conducting the review.

**Table 2:** Quality of individual meta-analysis assessed with AMSTAR-2.

	PICO	Protocol	Design	Search	Screening	Extraction	Exclusion	Inclusion	Bias	Funding	Statistics	Impact of bias	Discussion of bias	Heterogeneity	Publication bias	Conflict of interest
Barnicot et al., 2020	yes	no	yes	partial yes	yes	yes	no	partial yes	yes	no	no	no	no	no	yes	yes
Bighelli et al., 2018	yes	yes	no	partial yes	yes	yes	no	yes	yes	no	yes	yes	yes	yes	yes	yes
Bighelli et al., 2021	yes	yes	no	partial yes	yes	yes	no	no	yes	no	yes	yes	yes	yes	yes	yes
Bighelli et al., 2023	yes	yes	no	partial yes	yes	yes	no	partial yes	yes	no	yes	yes	yes	yes	yes	yes
Burns et al., 2014	yes	No	no	no	no	no	no	partial yes	partial yes	no	yes	no	no	yes	yes	yes
Jauhar et al., 2014	yes	no	no	partial yes	no	yes	yes	partial yes	yes	no	no	yes	yes	no	yes	yes
Jones, Hacker, Meaden et al., 2018	yes	yes	no	yes	yes	yes	yes	yes	yes	yes	yes	yes	yes	yes	no	yes
Jones, Hacker, Xia et al., 2018	yes	yes	no	yes	yes	yes	yes	yes	yes	yes	yes	yes	yes	yes	no	yes
Kennedy et al., 2017	yes	no	yes	partial yes	no	yes	yes	yes	yes	no	yes	yes	yes	yes	no	yes
Laws et al., 2018	yes	no	no	partial yes	no	no	yes	partial yes	no	no	no	no	no	no	yes	yes
Lincoln et al., 2008	yes	no	no	no	no	yes	no	no	no	no	yes	no	no	yes	yes	no
Lutgens et al., 2017	yes	partial yes	no	no	yes	no	no	yes	partial yes	no	yes	yes	yes	yes	yes	yes
Lynch et al., 2010	yes	no	no	no	no	yes	yes	partial yes	no	no	yes	yes	no	yes	no	yes
Mc Glanaghy et al., 2021	yes	yes	no	partial yes	yes	yes	no	partial yes	yes	no	yes	yes	yes	yes	no	yes
Mehl et al., 2015	yes	no	no	no	no	yes	no	partial yes	no	no	yes	no	no	yes	yes	yes
Naeem et al., 2016	yes	no	no	partial yes	yes	yes	no	partial yes	no	no	no	no	no	no	yes	no
Newton-Howes et al., 2013	yes	no	yes	no	no	yes	no	partial yes	no	no	no	no	no	no	yes	no
Pilling et al., 2002	yes	no	no	no	yes	no	no	no	no	no	yes	no	no	yes	no	no
Sarin et al., 2011	yes	no	no	partial yes	yes	yes	no	partial yes	yes	no	no	yes	yes	no	no	no
Todorovic et al., 2020	yes	no	no	partial yes	yes	yes	yes	yes	yes	no	yes	yes	yes	yes	no	yes
Turner, Reijnders et al., 2020	yes	no	no	partial yes	yes	yes	no	yes	partial yes	no	yes	yes	yes	yes	yes	yes
Turner, Burgers et al., 2020	yes	no	no	no	no	no	no	yes	yes	no	yes	yes	yes	yes	yes	yes
Turner, van der Gaag et al., 2014	yes	no	no	no	no	yes	no	partial yes	yes	no	no	yes	yes	yes	yes	yes
van der Gaag et al., 2014	yes	no	no	partial yes	no	no	yes	yes	yes	no	no	yes	yes	no	yes	yes
Velthorst et al., 2015	yes	no	no	no	no	no	no	partial yes	yes	no	yes	yes	yes	yes	yes	yes
Wykes et al., 2008	yes	no	yes	partial yes	yes	no	no	no	yes	no	yes	yes	yes	yes	yes	no

AMSTAR-2 scores across individual meta-analysis. Abbreviations: AMSTAR-2: assessment of multiple systematic reviews, PICO: Problem; Intervention; Comparison; Outcome.

### Grading of evidence

Details of the primary and secondary analysis are shown in Tables 3 and 4. 42.9% showed a significant result in favour of CBT and 57.1% of the comparisons were not significant. According to the hierarchy of evidence, none of the analysis demonstrated convincing or highly suggestive evidence in favour of CBT. 7.1% of the comparisons indicated suggestive evidence, while 35.7% comprised weak evidence. Other analyses were non-significant (57.1%). Results from meta-analyses providing insufficient data reported 286 comparisons, as shown in Supplemental Tables S2 and S3 24.8% (71/286) were significant in favour of CBT, and 75.2% (215/286) were not significant. All primary and secondary outcomes are visualized by forest plots per outcome and follow-up by Supplemental Figs. S1–S34 in the supplement.

**Table 3:** Overview of evidence used for current umbrella review ranked by class of evidence and year of publication.

Outcome	Authors and year	Intervention and control group	RCTs	Follow-up	Sample size	Hedges' G^1^	p-value	I^2^ (%)	PI 95 CI%	SSE/ESB/LS	CE
General psychopathology	Jauhar et al., 2014	CBT vs TAU (68%), Unknown (19%), BF (3%), PE (3%), SC (2%), SAT (2%), CR (1%), ST (1%), GS (1%)	34	EoT	2991	−0.33 (−0.47; −0.19)	0.42 × 10^−5^	67.93	Notnull	No/No/Yes	III
	Jones, Hacker, Meaden et al., 2018	CBT vs ST (72%), SAT (13%), NS-CG (8%), FT (7%)	9	>12 months	596	−0.21 (−0.40; 0.03)	0.03	14.32	Null	No/No/Yes	IV
	Jones, Hacker, Xia et al., 2018	CBT vs TAU (100%) (PANSS)	11	<6 months	962	−0.71 (−0.99; −0.43)	0.53 × 10^−6^	68.51	Null	Yes/No/No	IV
		CBT vs TAU (100%) (PANSS)	11	6–12 months	963	−0.36 (−0.59; −0.13)	<0.01	66.51	Null	Yes/No/No	IV
		CBT vs TAU (100%) (PANSS)	12	>12 months	1284	−0.29 (−0.50; 0.07)	0.01	76.63	Null	No/No/No	IV
		CBT vs TAU (100%) (BPRS)	5	<6 months	541	−0.63 (−1.04; −0.21)	0.003	73.54	Null	No/No/Yes	IV
		CBT vs TAU (100%) (BPRS)	3	>12 months	175	−0.97 (−1.50; −0.44)	0.35 × 10^−3^	61.96	Null	No/No/Yes	IV
	Todorovic et al., 2020	CBT vs TAU (86%), ST (7%), BF (4%), SC + PE (3%)	4	EoT	524	−0.33 (−0.70; 0.04)	0.08	43.11	Null	No/Yes/No	NS
		CBT vs TAU (92%), BF (4%), SC + PE (4%)	3	6–12 months	470	−0.10 (−0.28; 0.09)	0.30	0	Null	No/No/No	NS
	Barnicot et al., 2020	CBT vs TAU (39%), PE (33%), ND-SC (21%), NS-SC + PE (7%)	5	EoT	284	−0.04 (−0.56; 0.48)	0.87	73.90	Null	No/Yes/Yes	NS
	Jones, Hacker, Meaden et al., 2018	CBT vs ST (56%), SP + PT (18%), SC + PE (14%), E− ST (12%)	6	<6 months	568	−0.23 (−0.48; 0.01)	0.06	75.40	Null	Yes/No/No	NS
		CBT vs NS-C (44%), Group SST (42%), SC + PE (14%)	3	<6 months	162	0.04 (−0.27; 0.35)	0.80	6.42	Null	No/No/No	NS
		CBT vs ST (100%) (BPRS)	3	6–12 months	270	−0.35 (−0.72; 0.02)	0.06	49.70	Null	No/Yes/Yes	NS
	Jones, Hacker, Xia et al., 2018	CBT vs TAU (100%) (BPRS)	3	6–12 months	199	−0.30 (−0.62; 0.02)	0.07	20.22	Null	No/No/No	NS
	Newton et al., 2011	CBT vs SC (49%), PE (16%), BF (15%), ST (13%), RT (7%)	9	EoT	602	0.040 (−0.30; 0.38)	0.82	70.93	Null	No/No/No	NS
	Lynch et al., 2010	CBT vs SC (46%), PE (16%), Group BF (15%), SAT (10%), RT (7%), ST (6%)	9	EoT	601	−0.09 (−0.26; 0.08)	0.30	20.97	Null	Yes/No//No	NS
Positive symptoms	Jauhar et al., 2014	TAU (60%), Unknown (21%), PE (5%), GF-SC (3%), BF (2%), SAT (2%), SC (2%), RS (2%), CR (2%), ST (1%), GS (0%)	33	EoT	2452	−0.26 (−0.37; −0.14)	0.20 × 10^−4^	52.44	Null	No/No/Yes	III
	Todorovic et al., 2020	TAU (86%), ST (7%), BF (3.5%), SC + PE (3.5%)	4	EoT	525	−0.33 (−0.50; −0.16)	0.19 × 10^−3^	0	Null	No/No/Yes	IV
		CBT vs TAU (92%), BF (4%), SC + PE (4%)	3	6–12 months	470	−0.20 (−0.38; −0.02)	0.03	0	Null	No/No/No	IV
	Bighelli et al., 2018	CBT vs TAU (100%)	18	EoT	1464	−0.28 (−0.40; −0.16)	0.45 × 10^−5^	20.19	NotNull	No/No/No	IV
	Jones, Hacker, Meaden et al., 2018	CBT vs ST (75%), PE (16%), SC (9%) (PANSS)	6	6–12 months	497	−0.25 (−0.42; −0.07)	0.67 × 10^−2^	0	Null	No/No/No	IV
		ST (53%), SAT (13%), E-ST (11%), GF-SC (9%), PE (8%), FI (7%)	9	>12 months	602	−0.30 (−0.46; −0.13)	0.34 × 10^−3^	0	Not Null	No/No/Yes	IV
	Barnicot et al., 2020	CBT vs TAU (54%), PE (46%)	3	EoT	203	−0.37 (−0.84; 0.09)	0.11	52.84	Null	No/Yes/Yes	NS
	Jones, Hacker, Meaden et al., 2018	CBT vs ST (35%), GF-SC (19%), PE (13%), SP + PT (11%), SAT (9%), E-ST (8%), SC (5%)	11	<6 months	883	−0.11 (−0.25; 0.04)	0.16	3.06	Null	No/No/No	NS
Negative symptoms	Jones, Hacker, Meaden et al., 2018	ST (58%), SAT (14%), E-ST (12%), PE (9%), FI (7%)	8	>12 months	548	−0.20 (−0.37; −0.03)	0.02	0	Null	Yes/No/Yes	IV
	Lutgens et al., 2017	CBT vs TAU (54%), ST (24%), TAU + WL (14%), BF (4%), SC + PE (3%), PE (2%)	16	EoT	1473	−0.32 (−0.53; −0.12)	0.20 × 10^−2^	71.76	Null	No/No/Yes	IV
	Jauhar et al., 2014	TAU (59%), CR (10%), Unknown^1^ (8%), BF (6%), PE (5%), GFT (3%), SAT (2%), SC (2%), RS (2%), ST (2%), GS (1%)	34	EoT	2354	−0.13 (−0.24; −0.02)	0.02	51.28	Null	No/No/Yes	IV
	Todorovic et al., 2020	CBT vs TAU (86%), ST (7%), BF (4%), SC + PE (3%)	4	EoT	529	−0.10 (−0.27; 0.07)	0.27	0	Null	No/No/No	NS
		CBT vs (TAU 92%), BF (4%), SC + PE (4%)	3	6–12 months	472	0.02 (−0.16; 0.20)	0.83	0	Null	No/No/No	NS
	Turner, Reijnders et al., 2020	CBT vs Unknown (45%), SC (29%), BF (18%), CR (5%), PE (3%)	10	EoT	821	0.05 (−0.08; 0.19)	0.45	0	Null	No/No/No	NS
	Jones, Hacker, Xia et al., 2018	CBT vs TAU (100%)	4	<6 months	231	−0.65 (−1.66; 0.37)	0.21	91.43	Null	No/No/No	NS
	Jones, Hacker, Meaden et al., 2018	CBT vs ST (64%), PE (23%), SC (13%) (PANSS)	4	6–12 months	359	−0.15 (−0.36; 0.06)	0.17	0	Null	Yes/No/No	NS
		CBT vs ST (40%), PE (21%), TAU (17%), SAT (14%), SC (8%) (PANSS)	7	<6 months	581	−0.04 (−0.21; 0.13)	0.63	7.72	Null	No/No/No	NS
	Velthorst et al., 2015	CBT vs TAU (49%), PE (11%), WL (9%), E-ST (8%), WL + TAU (6%), SC (5%), BF (5%), E-TAU (4%), Unknown (3%)	13	3–6 months	895	0.21 (−0.05; 0.47)	0.11	73.11	Null	No/No/No	NS
		CBT vs TAU (72%), BF (15%), GFSC (6%), E− ST (7%)	10	9–12 months	916	−0.008 (−0.20; 0.19)	0.94	52.49	Null	No/No/No	NS
		CBT vs TAU (60%), BF (9%), PE (6%), unknown (5%), ETAU (4%), WL (3%), GFSC (3%), E-ST (3%), WL + TAU (3%), SC (2%), ST (2%)	28	EoT	2067	0.12 (−0.03; 0.27)	0.13	64.63	Null	Yes/No/No	NS
Delusions	Turner, Burger et al., 2020	CBT vs TAU (76%, SC (20%), PE (3%), ET (1%)	27	EoT	2169	0.36 (0.22; 0.51)	0.15 × 10^−5^	50.47	Null	Yes/No/No	III
	Mehl et al., 2015	CBT vs TAU (100%)	13	Eot	1094	0.26 (0.08; 0.45)	0.56 × 10^−2^	38.53	Null	Yes/No/No	IV
	Van der Gaag et al., 2014	CBT vs TAU (54%), SC (34%), SAT (9%), AC (3%)	11	Eot	768	0.36 (0.08; 0.64)	0.01	57.38	Null	No/No/Yes	IV
	Mehl et al., 2015	SC (50.8%), SAT (14.6%), FT (10.8%), ST (8.8%), PE (5.8%), AC (4.6%), PS (4.6%)	8	EoT	659	0.15 (−0.12; 0.42)	0.29	38.55	Null	No/No/No	NS
		CBT vs SC (60%), SAT (17%), FT (13%), ST (10%)	5	<9 months	578	−0.04 (−0.25; 0.17)	0.69	0	Null	No/No/No	NS
		CBT vs TAU (100%)	12	<12 months	1391	0.16 (−0.03; 0.34)	0.10	40.71	Null	No/No/No	NS
Hallucinations	Turner, Burger et al., 2021	CBT vs TAU (72%), SC (24%), PE (3%), WL (1%)	28	EoT	2388	0.32 (0.19; 0.46)	0.41 × 10^−5^	45.14	Null	No/No/Yes	III
	Jauhar et al., 2014	CBT vs TAU (42%), unknown (30%), ST (8%), SAT (6%), SC (6%), BF (5%), PE (3%)	15	EoT	778	−0.34 (−0.62; −0.06)	0.02	71.17	Null	No/No/No	IV
	Van der Gaag et al., 2014	CBT vs TAU (60%), SC (32%), SAT (8%)	13	EoT	822	0.44 (0.27; 0.61)	0.55 × 10^−6^	0	notNull	No/No/Yes	IV

1 In some cases the summary estimates from our analysis differed one-hundredth decimal compared to the original findings. We used the data provided by the report of the meta-analysis and expect that variation between estimates adopted from the primary RCTs and estimates provided in the meta-analysis have caused this slight and clinically irrelevant variation.

**Table 4:** Grading the evidence of secondary outcomes ranked by class of evidence and year of publication.

Outcome	First author and year	Intervention and control group	RCTs	Follow-up	Sample size	Hedges' G^1^	p-value	I^2^ (%)	PI 95CI%	SSE/ESB/LS	CE
Affective symptoms	Jones, Hacker, Meaden et al., 2018	CBT vs SP-PT (25%), PE (24%), SAT (20%), E-ST (17%), SC (12%), ST (3%)	6	<6 months	400	−0.24 (−0.67; 0.19)	0.28	81.74	Null	No/No/Yes	NS
		CBT vs PE (42%), E-ST (34%), SC (24%) (PANSS)	3	6–12 months	194	−0.12 (−0.72; 0.48)	0.69	77.28	Null	No/No/No	NS
		CBT vs ST (40%), SAT (20%), E-ST (17%), PE (13%), FI (10%)	7	>12 months	379	−0.14 (−0.36; 0.08)	0.21	14.13	Null	No/No/No	NS
Anxiety	Jones, Hacker, Xia et al., 2018	CBT vs TAU (100%) (BAI)	3	>12 months	335	0.12 (−0.10; 0.34)	0.27	0	Null	No/No/No	NS
Depressive symptoms	–										
Rehospitalisation	Jones, Hacker, Meaden et al., 2018	CBT vs PE (84%), ST (16%)	3	6–12 months	78	−0.08 (−0.31; 0.14)	0.48	0	Null	Yes/No/No	NS
		CBT vs PE (34%), ST (21%), CR (18%), BF (14%), E− ST (7%), RT (6%)	8	>12 months	595	−0.02 (−0.10; 0.06)	0.64	0	Null	No/No/No	NS
	Jones, Hacker, Xia et al., 2018	CBT vs TAU (100%)	6	>12 months	648	−0.12 (−0.28; 0.03)	0.11	0	Null	No/No/No	NS
Relapse	Jones, Hacker, Xia et al., 2018	CBT vs TAU (100%)	5	6–12 months	667	−0.35 (−0.52; −0.18)	0.60 × 10^−4^	0	Not Null	Yes,/No/Yes	IV
	Jones, Hacker, Meaden et al., 2018	CBT vs TAU (51%), SC (17%), RT (17%), FI (15%)	5	>12 months	375	0.02 (−0.09; 0.13)	0.72	0	Null	No/No/No	NS
	Jones, Hacker, Xia et al., 2018	CBT vs TAU (100%)	13	>12 months	1538	−0.14 (−0.29; 0.001)	0.05	51.47	Null	Yes/No/No	NS
	Lynch et al., 2010	CBT vs TAU (51%), SC (29%), ST (11%), PE (9%)	8	6–36 months	976	0.05 (−0.18; 0.29)	0.66	41.10	Null	No/No/Yes	NS
Functioning and distress	Bighelli et al., 2023	CBT vs TAU (64%), inactive control (8%), ST (10%), CR (9%), WL (6%), FI (1%), psychodynamic therapy (1%), PE (1%)	30	EoT	2657	0.24 (0.11; 0.38)	0.50 × 10^−3^	58.18	Null	No/No.No	III
	Laws et al., 2018 (distress)	CBT vs TAU (83%), unknown (11%), WL (6%)	8	EoT	573	0.37 (0.06; 0.67)	0.02	58.76	Null	No/No/No	IV
		CBT vs TAU (61%), CR (14%), ST (9%), medication (5%), SAT (5%), BF (4%), PE (2%), WL (1%)	26	EoT	1704	0.25 (0.10; 0.40)	0.76 × 10^−3^	54.50	Null	No/No/No	IV
	Bighelli et al., 2023	CBT vs TAU (100%)	19	EoT	1682	0.34 (0.14. 0.53)	0.65 × 10^−3^	63.68	Null	Yes/No/No	IV
	Bighelli et al., 2023	CBT (third-wave, mindfulness (63%), ACT (9%), MCT (28%)) vs mixed control	20	EoT	1391	0.58 (0.36; 0.80)	0.29 × 10^−6^	71.94	Null	No/No/Yes	IV
	Bighelli et al., 2023	CBT (third-wave, mindfulness (100%) vs mixed control	12	EoT	989	0.71 (0.45; 0.96)	0.57 × 10^−7^	68.70	Null	No/No/Yes	IV
	Bighelli et al., 2023	CBT (third-wave, MCT (100%)) vs mixed control	7	EoT	155	0.45 (0.01; 0.88)	0.04	67.96	Null	No/No/No	IV
	Laws et al., 2018	CBT vs TAU (61%), CR (14%), ST (9%), medication (5%), SAT (5%), BF (4%), PE (2%), WL (1%)	16	3–18 months	938	0.10 (−0.07; 0.27)	0.23	34.84	Null	No/No/No	NS
	Jones, Hacker, Xia et al., 2018	CBT vs TAU (100%)	5	>12 months	446	0.15 (−0.14; 0.44)	0.32	44.47	Null	No/No/No	NS
		CBT vs TAU (100%)	5	6–12 months	482	0.22 (−0.12; 0.55)	0.21	59.81	Null	No/Yes/No	NS
	Jones, Hacker, Meaden et al., 2018	CBT vs NS-C (55%), SAT (45%)	3	>12 months	224	0.22 (−0.29; 0.73)	0.02	72.44	Null	No/No/No	NS
	Bighelli et al., 2023	CBT vs goal-focused supportive contact (56%), SAT (27%), ACT (17%)	4	EoT	229	0.01 (−0.40; 0.43)	0.96	58.23	Null	No/No/No	NS
Quality of life	Laws et al., 2018	CBT vs TAU (62%), PE (15%), WL (8%), medication (8%), TAU + medication (7%)	10	EoT	626	0.04 (−0.12; 0.19)	0.64	0	Null	No/No/No	NS
Social functioning	Barnicot et al., 2020	CBT vs TAU (65%), ND-SC (35%)	3	EoT	175	0.66 (−0.07; 1.39)	0.08	69.71	Null	No/No/Yes	NS

Abbreviations Tables 2 and 3: PI: prediction interval, SSE: small study effect, ESB: excess significance bias, LS: largest study, CE: class of evidence, RCT: randomized controlled trial, EoT: end of therapy, ES: effect size, CBT: cognitive behavioral therapy, TAU: treatment as usual, PE: psychoeducation, NS-SC + PE: supportive counseling + psychoeducation, ST: supportive therapy, SC: supportive counseling, SAT: Social Activity Therapy, PANSS: Positive and Negative Syndrome Scale, SST: Social Skills Training, BPRS: Brief Psychiatric Rating Scale, BF: befriending, RT: Recreational Therapy, CR: Cognitive remediation, WL: waiting list, AT: active treatment, BAI: Beck Anxiety Inventory, ND-SC: Non-Directive Supportive Counseling, NS-SC: , FI: , FT: family intervention, GS: group support, ET: exposure therapy, AC: Attention (Placebo) Control, NS-CG: Non-specific counselling group, SP + PT: Standard psychological support + pharmacological therapy, NS-C: Non-specific counselling, E-ST: enhanced supportive therapy, GF-SC: Goal-focused supportive contact, RS: Recreation and Support, GFT: goal focused therapy, Positive and Negative Syndrome Scale, BPRS: Brief Psychiatric Rating Scale, ETAU: enriched treatment as usual.

1 In some cases the summary estimates from our analysis differed one-hundredth decimal compared to the original findings. We used the data provided by the report of the meta-analysis and expect that variation between estimates adopted from the primary RCTs and estimates provided in the meta-analysis have caused this slight and clinically irrelevant variation.

### Primary outcome

43.8% of the correlations involving general psychopathology were significant. One comparison (6.3%) showed suggestive evidence in favour of CBT vs mostly treatment as usual (TAU) at end of treatment (EoT). 37.5% showed weak evidence at various follow-up assessments, mostly CBT vs TAU or supportive therapy (ST). 56.3% of the comparisons were non-significant at variable follow-up, and control groups consisted of TAU, ST, and active components. Of these, 55.6% of the comparisons were sufficiently powered to detect an ES of 0.3 and all comparisons were sufficiently powered to detect an ES of 0.5 or 0.8. Details of the power analyses are shown in Table 5.

**Table 5:** Power-analysis of non-significant results.

Outcome	First authors and year	Intervention and control group	RCTs	Follow-up	Sample size	Hedges' G	p-value	ES 0.3	ES 0.5	ES 0.8
General psychopathology	Barnicot et al., 2020	CBT vs TAU (39%), PE (33%), ND-SC (21%), NS-SC + PE (7%)	5	EoT	284	−0.04 (−0.56; 0.48)	0.87	71	99	100
	Jones, Hacker, Meaden et al., 2018	CBT vs ST (56%), SP + PT (18%), SC + PE (14%), E− ST (12%)	6	<6 months	568	−0.23 (−0.48; 0.01)	0.06	95	100	100
		CBT vs NS-C (44%), Group SST (42%), SC + PE (14%)	3	6–12 months	162	0.04 (−0.27; 0.35)	0.80	48	89	99
		CBT vs ST (100%) (BPRS)	3	<6 months	270	−0.35 (−0.72; 0.02)	0.06	69	98	100
	Jones, Hacker, Xia et al., 2018	CBT vs TAU (100%) (BPRS)	3	6–12 months	199	−0.30 (−0.62; 0.02)	0.07	56	94	100
	Lynch et al., 2010	CBT vs SC (46%), PE (16%), Group BF (15%), SAT (10%), RT (7%), ST (6%)	9	EoT	601	−0.09 (−0.26; 0.08)	0.30	96	100	100
	Newton et al., 2011	CBT vs SC (49%), PE (16%), BF (15%), ST (13%), RT (7%)	9	EoT	602	0.04 (−0.30; 0.38)	0.82	96	100	100
	Todorovic et al., 2020	CBT vs TAU (86%), ST (7%), BF (4%), SC + PE (3%)	4	EoT	524	−0.33 (−0.70; 0.04)	0.08	93	100	100
		CBT vs TAU (92%), BF (4%), SC + PE (4%)	3	6–12 months	470	−0.10 (−0.28; 0.09)	0.30	90	100	100
Positive symptoms	Jones, Hacker, Meaden et al., 2018	CBT vs ST (35%), GF-SC (19%), PE (13%), SP + PT (11%), SAT (9%), E-ST (8%), SC (5%)	11	<6 months	883	−0.11 (−0.25; 0.04)	0.16	99	100	100
	Barnicot et al., 2020	CBT vs TAU (54%), PE (46%)	3		203	−0.37 (−0.84; 0.09)	0.11	57	94	100
Negative symptoms	Jones, Hacker, Meaden et al., 2018	CBT vs ST (64%), PE (23%), SC (13%) (PANSS)	4	6–12 months	359	−0.15 (−0.36; 0.06)	0.17	95	100	100
		CBT vs ST (40%), PE (21%), TAU (17%), SAT (14%), SC (8%) (PANSS)	7	<6 months	581	−0.04 (−0.21; 0.13)	0.63	14	32	54
	Todorovic et al., 2020	CBT vs TAU (86%), ST (7%), BF (4%), SC + PE (3%)	4	EoT	529	−0.010 (−0.27; 0.07)	0.27	93	100	100
	Turner, Reijnders et al., 2020	CBT vs Unknown (45%), SC (29%), BF (18%), CR (5%), PE (3%)	10	Eot	821	0.05 (−0.08; 0.19)	0.45	99	100	100
	Velthorst et al., 2015	CBT vs TAU (49%), PE (11%), WL (9%), E-ST (8%), WL + TAU (6%), SC (5%), BF (5%), ETAU (4%), Unknown (3%)	13	3–6 months	895	0.21 (−0.05; 0.47)	0.11	63	97	100
		CBT vs TAU (72%), BF (15%), GFSC (6%), E− ST (7%)	10	9–12 months	916	−0.008 (−0.20; 0.19)	0.94	100	100	100
	Todorovic et al., 2020	CBT vs (TAU 92%), BF (4%), SC + PE (4%)	3	6–12 months	472	0.02 (−0.16; 0.20)	0.83	90	100	100
	Jones, Hacker, Xia et al., 2018	CBT vs TAU (100%)	4	<6 months	231	−0.65 (−1.66; 0.37)	0.21	76	99	100
	Velthorst et al., 2015	CBT vs TAU (60%), BF (9%), PE (6%), unknown (5%), ETAU (4%), WL (3%), GFSC (3%), E-ST (3%), WL + TAU (3%), SC (2%), ST (2%)	28	EoT (secondary)	2067	0.12 (−0.03; 0.27)	0.13	100	100	100
Delusions	Mehl et al., 2015	CBT vs SC (50.8%), SAT (14.6%), FT (10.8%), ST (8.8%), PE (5.8%), AC (4.6%), PS (4.6%)	8	EoT	659	0.15 (−0.12; 0.42)	0.29	96	100	100
		CBT vs SC (60%), SAT (17%), FT (13%), ST (10%)	5	<9 months	578	−0.04 (−0.25; 0.17)	0.69	94	100	100
		CBT vs TAU (100%)	12	<12 months	1391	0.16 (−0.03; 0.34)	0.10	100	100	100
Affective symptoms	Jones, Hacker, Meaden et al., 2018	CBT vs SP-PT (25%), PE (24%), SAT (20%), E-ST (17%), SC (12%), ST (3%)	6	<6 months	400	−0.24 (−0.67; 0.19)	0.28	55	93	100
		CBT vs PE (42%), E-ST (34%), SC (24%) (PANSS)	3	6–12 months	194	−0.12 (−0.72; 0.48)	0.69	85	100	100
		CBT vs ST (40%), SAT (20%), E-ST (17%), PE (13%), FI (10%)	7	>12 months	379	−0.14 (−0.36; 0.08)	0.21	83	100	100
Anxiety	Jones, Hacker, Xia et al., 2018	CBT vs TAU (100%) (BAI)	3	>12 months	335	0.12 (−0.10; 0.34)	0.27	78	100	100
Rehospitalisation	Jones, Hacker, Meaden et al., 2018	CBT vs PE (84%), ST (16%)	3	6–12 months	78	−0.08 (−0.31; 0.14)	0.48	24	55	83
		CBT vs PE (34%), ST (21%), CR (18%), BF (14%), E− ST (7%), RT (6%)	8	>12 months	595	−0.02 (−0.10; 0.06)	0.64	89	100	100
	Jones, Hacker, Xia et al., 2018	CBT vs TAU (100%)	6	>12 months	648	−0.12 (−0.28; 0.03)	0.11	97	100	100
Relapse	Jones, Hacker, Meaden et al., 2018	CBT vs TAU (51%), SC (17%), RT (17%), FI (15%)	5	>12 months	375	0.02 (−0.09; 0.131)	0.72	83	100	100
	Jones, Hacker, Xia et al., 2018	CBT vs TAU (100%)	13	>12 months	1538	−0.14 (−0.29; 0.001)	0.052	100	100	100
	Lynch et al., 2010	CBT vs TAU (51%), SC (29%), ST (11%), PE (9%)	8	6–36 months	976	0.05 (−0.18; 0.29)	0.66	100	100	100
Social functioning	Barnicot et al., 2020	CBT vs TAU (65%), ND-SC (35%)	3	EoT	175	0.66 (−0.07; 1.39)	0.08	51	91	100
Functioning and distress	Laws et al., 2018	CBT vs TAU (61%), CR (14%), ST (9%), medication (5%), SAT (5%), BF (4%), PE (2%), WL (1%)	16	3–18 months	938	0.10 (−0.07; 0.27)	0.23	100	100	100
	Jones, Hacker, Xia et al., 2018	CBT vs TAU (100%)	5	>12 months	446	0.15 (−0.14; 0.44)	0.32	88	100	100
		CBT vs TAU (100%)	5	6–12 months	482	0.22 (−0.12; 0.55)	0.21	91	100	100
	Bighelli et al., 2023	CBT vs goal-focused supportive contact (56%), SAT (27%), ACT (17%)	4	EoT	229	0.01 (−0.40; 0.43)	0.96	62	96	100
Quality of life	Laws et al., 2018	CBT vs TAU (62%), PE (15%), WL (8%), medication (8%), TAU + medication (7%)	10	EoT	626	0.04 (−0.12; 0.19)	0.64	96	100	100

Abbreviations: PI: prediction interval, SSE: small study effect, ESB: excess significance bias, LS: largest study, CE: class of evidence, RCT: randomized controlled trial, EoT: end of therapy, ES: effect size, CBT: cognitive behavioral therapy, TAU: treatment as usual, PE: psychoeducation, NS-SC + PE: , ST: supportive therapy, SC: supportive counseling, SAT: Social Activity Therapy, PANSS: Positive and Negative Syndrome Scale, SST: Social Skills Training, BPRS: Brief Psychiatric Rating Scale, BF: befriending, RT: Recreational Therapy, CR: Cognitive remediation, WL: waiting list, AT: active treatment, BAI: Beck Anxiety Inventory, ND-SC: Non-Directive Supportive Counseling, NS-SC: , FI: , FT: family intervention, GS: group support, ET: exposure therapy, AC: Attention (Placebo) Control, NS-CG: Non-specific counselling group, SP + PT: Standard psychological support + pharmacological therapy, NS-C: Non-specific counselling, E-ST: enhanced supportive therapy, GF-SC: Goal-focused supportive contact, RS: Recreation and Support, GFT: goal focused therapy.

Concerning positive symptoms, 12.5% of the comparisons showed suggestive evidence in favor of CBT vs mostly TAU at EoT, 62.5% showed weak evidence at variable follow-up compared to TAU and ST. 25% of the comparisons were non-significant at the EoT or less than 6 months follow-up. Control groups consisted of TAU and ST. Of the non-significant comparisons the study of Jones, Hacker, Meaden et al. 2018 was sufficiently powered to detect an ES of 0.3. The study of Barnicot et al., 2020 was only sufficiently powered to detect an ES of 0.5 and 0.8.

Regarding delusions, 16.7% presented suggestive evidence of CBT vs TAU at the EoT. 33.3% showed weak evidence at EoT in favour of CBT compared to mixed control groups and 50% of the analyses were non-significant at follow-up compared to TAU and active treatment. Effect sizes ranged from 0.261 to 0.363. Hallucinations showed suggestive evidence in 33.3% at EoT in favour of CBT vs mostly TAU. 66.7% showed weak evidence at the EoT in favour of CBT compared to mixed control groups. All non-significant findings were sufficiently powered.

No comparisons indicated suggestive evidence for negative symptoms. 25% showed weak evidence in favour of CBT at EoT and at >12 months follow-up, with control groups consisting of TAU and ST. 75% of the correlations were non-significant at follow-up EoT or 12 months compared to TAU. Of the non-significant findings 66.7% were sufficiently powered to detect an effect size of 0.3 and 88.9% of the comparisons ;were powered to find an effect size of 0.5 or 0.8.

### Secondary outcomes

Comparisons of the outcome relapse and rehospitalisation demonstrated weak evidence in 14.3% at 6–12 months follow-up compared to TAU. 85.7% of the comparisons were non-significant of CBT vs active treatment or TAU, at follow-up of 6–12 months and 12–36 months. All non-significant findings were sufficiently powered in 85.7% of the cases, 100% for effect size of 0.8. With regard to functioning and distress, 8.3% showed suggestive evidence in favour of CBT, 50.0% demonstrated weak evidence in favour of CBT. 33.3% were non-significant comparisons. All analyses were sufficiently powered.

Grading evidence of other secondary outcomes showed non-significant comparisons for treatment of affective symptoms, anxiety, quality of life, or social functioning. Non-significant findings of affective symptoms were powered in 66.7% of the cases to find an effect size of 0.3, and sufficiently powered in 100% of the comparisons to find an effect size of 0.5 and 0.8. Non-significant findings with regard to quality of life were sufficiently powered.

### Study parameters

Heterogeneity measured by I^2^ was less than 50% in 50.0% of the found comparisons. Small-study effects were found in 21.4% of the findings and excess significance bias was found in 15.7% of the comparisons. 31.4% of the largest RCTs were significant and 24.0% of the prediction intervals excluded zero. Small-study effects were found in general psychopathology in 25%, 25% in negative symptoms, 33.3% in delusions, none in hallucinations. Excess-significance bias was found in 18.8% of the findings in general psychopathology, 12.5% in positive symptoms, and 0% in negative symptoms, delusions, and hallucinations. The largest study was significant in 37.5% of the comparisons for general psychopathology, 50% for positive symptoms, and 25% for negative symptoms. With regard to delusions 16.7% of the largest study was significant and in 67.7% of the outcome hallucinations. Prediction intervals excluding zero were found in general psychopathology (6.7%), positive symptoms (25%), hallucinations (33.3%), relapse (25%), none in negative symptoms, delusions or other secondary outcomes. Generally, high parameters of SSE or ESB were not found in secondary outcomes.

### Sensitivity analysis

In Supplemental Table S4 comparisons between CBT vs any control group are shown that were based on more than 20 RCTs. Results are partly similar to the primary findings. In the sensitivity analysis, at the EoT the following outcome parameters significantly improved in favor of CBT: general psychopathology (Hedges' G −0.33 (−0.47; −0.19)), positive symptoms (Hedges' G −0.26 (−0.37; −0.14)) delusions (Hedges' G 0.36 (0.22; 0.51)), hallucinations (Hedges' G 0.32 (0.19; 0.46)), and functioning (Hedges' G 0.24–0.25 (0.10–0.40)). One analysis showed improvement of negative symptoms in favor of CBT (Hedges' G −0.13 (−0.24; −0.02)), while another analysis showed a non-significant finding (Hedges’ G 0.12 (−0.03; 0.27)). There were not more than 20 RCTs available for outcomes such as relapse, affective symptoms, depressive symptoms, anxiety, rehospitalisation, quality of life, or social functioning.

### Other supplementary data

Comparisons from meta-analyses providing insufficient data for analyses showed that 35.2% of the comparisons in general psychopathology were significant, 33.3% were significant in positive symptoms, 28.6% in negative symptoms, 66.7% in delusions, and 60% were significant in hallucinations. Secondary outcomes demonstrated that 66.7% of the comparisons for affective symptoms were significant, none were significant for depressive symptoms, 50% for rehospitalisation, and 10.8% for relapse. Comparisons investigating functioning and distress had 19.2% significant comparisons, 60% in quality of life, and none in suicide were significant.

## Discussion

The present study employed an umbrella approach to investigate evidence for the effectiveness of CBT by providing an extensive overview of meta-analytical data and a stratification of evidence on relevant clinical outcomes. Overall, 16 meta-analyses provided 70 comparisons of CBT vs any other control group and showed no convincing or highly suggestive evidence in favor of CBT. Concerning core symptoms of SSD, we found suggestive evidence in a minority of the comparisons and weak evidence in the majority of the comparisons supporting CBT for the treatment of general psychopathology, positive symptoms (delusions and hallucinations) at the end of treatment. In addition, we also found suggestive evidence for the improvement of functioning at the end of treatment. Results from current umbrella review also suggested that CBT has limited or no effect on other important outcomes such as psychotic relapse, negative symptoms, and quality of life, not in line with recommendations made by American, British, and Dutch clinical guidelines.^1^, ^2^, ^3^ These findings were confirmed by sensitivity analysis.

This umbrella review partly confirms previous research carried out during the past decades on the clinical effect of CBT compared to treatment as usual for the improvement of clinically relevant symptoms and functioning of psychosis. Findings indicate that CBT may reduce disturbing symptoms such as paranoid delusions or hearing voices by the end of treatment. The same accounts for the effect of CBT on functioning, in which a small effect was found at the end of treatment in favor of CBT (Table 3 and Supplemental Table S3). However, findings changed to weak or non-significant evidence at follow-up, while they were well-powered and in rather large samples. This suggests that a long-term sustainable effect of CBT may be missing on group level. The effectiveness for quality of life or social functioning is not convincing. Merely one meta-analysis of 10 RCTs demonstrated no effect of CBT on quality of life, while a meta-analysis by Bighelli et al., 2018 (Supplemental Table S3) showed a significant effect in a smaller sample.^20^ Our findings furthermore suggest that there is no evidence supporting CBT for the prevention of psychotic relapses, as the majority of the comparisons were non-significant and well-powered.

Not in line with several clinical guidelines, current study observed that approximately 75% of the comparisons focusing on improvement of negative symptoms were non-significant, most of the analyses were well-powered studies and the largest comparison included more than 1000 persons. This suggests that CBT has no clinically relevant effect on negative symptoms and may therefore not be recommended for this indication. This conclusion is comparable to a previous meta-analysis by Fusar-Poli and colleagues investigating placebo-controlled studies for the improvement of negative symptoms. We agree with the authors concluding that currently no effective treatment for negative symptoms is available.^21^ Negative symptoms remain a substantial clinical and research challenge and further research should aim to elucidate its pathophysiology and therapy.^22^

Of note, the current study focused on CBT and not on other potentially effective treatments for psychosis such as metacognitive training or cognitive remediation.^23^^,^^24^ In fact, CBT forms a broad collection of different approaches such as enhanced coping methods, homework, imagery, change strategies or mindfulness all captured within the individual case-formulation. The effect of these individualized interventions on broad outcomes measures (e.g. PANSS or social functioning) may be difficult to apprehend.^25^ More emphasis on individualized outcome parameters (e.g. stress caused by hallucinations) could potentially lead to higher effect sizes. Two previous umbrella reviews concerning the effectiveness of psychosocial treatment in schizophrenia reported effect sizes in the small to medium range for CBT in the treatment of general symptoms.^26^^,^^27^ The authors used a more global approach without a detailed analyses of study parameters, description of sample size and ranking of evidence on the effectiveness of CBT. Therefore, their findings could not be used to evaluate inconsistent results between meta-analyses. From a wider perspective, the umbrella approach was also used to investigate the efficacy of psychotherapy in RCTs and psychosocial treatment of autism spectrum disorders. Both studies found a large variety of evidence from class I to non-significant.^18^^,^^28^ In comparison, we found no evidence in the highest classes and only a small number of comparisons that fell into class III.

Assessment of study parameters demonstrated a general lack of excess significance bias, small study effects, and low heterogeneity in the majority of found comparisons. Moreover, only a minority of the comparisons was affected by small study effects and excess significance bias. The prediction interval generally included zero and the sign for largest study significance was mostly negative. Therefore, we conclude that current findings have a high validity and indicating that they may actually represent true findings. Previous research concerning small-study effects and excess significance bias found similar results in RCTs with CBT for other psychiatric conditions.^18^

On a more global level we evaluated methodological quality of meta-analyses using the AMSTAR-2. We found specific shortcomings in the explanation of the selection of study design in the review, described reasons for exclusion, performing the search in duplicate, and reporting of funding were done in less than 60% of the meta-analyses. Other important areas such as addressing heterogeneity, impact and discussion of bias, performing data-extraction in duplicate, and characterisation of the included population were done more frequently (>60%). We used a different approach in interpreting the AMSTAR-2 scores compared to previous umbrella reviews examining psychosocial treatments for schizophrenia. For example. Solmi et al. found that 1.3% of the included meta-analyses had high quality, while the other were rated medium or low quality.^11^^,^^27^ Overall, we found strengths and limitations on several methodological areas of included meta-analyses. We therefore advise future researchers to study the AMSTAR-2 when designing a meta-analysis.

The rise of the umbrella approach is evident and the number of publications using this methodology is increasing. Several authors highlighted advantages and shortcomings of this particular technique. Papatheodorou^29^ and Fusar-Poli et al.^12^ outlined benefits in terms of providing an overview of inconsistent scientific findings and ranking evidence. On the other side of the debate, Schlesinger^30^ et al. and Gianfredi et al.^31^ draw its shortcomings as the main focus is on statistical significance, the employment of arbitrary cutoffs, and lack of measures of clinical relevance. We concur with some of these limitations and we think that the inclusion of parameters such as number needed to treat may enhance its interpretation. Furthermore, the large majority of comparisons in our study was based on less than 1000 patients receiving CBT, making it impossible to be classified as class III or higher. Despite that, the greater part of the found comparisons in our study was non-significant on several outcome measures and may therefore never be classified as IV or higher. It has been recommended to assert the instrument Grading of Recommendations, Assessment, Development and Evaluations (GRADE) instead of the class of evidence, as it relies more on certainty of evidence and precision of findings. However, the GRADE includes a substantial degree of subjectivity in the classification since it is mainly dependent on judgment of the rater.^32^

We should interpret our findings in light of several other limitations. First and foremost, a large proportion of the meta-analyses provided insufficient data to repeat the meta-analysis, this has negatively impacted the precision our findings. Secondly, we did not contact individual authors for any missing data, making the review dependent on published reports. Thirdly, some items of the AMSTAR-2 may not reflect methodological quality in itself. For example, item 13 of the AMSTAR-2 requires the meta-analyses to only include RCTs with low risk of bias or if RCTs with moderate or high risk of bias were included the authors should provide a discussion of the impact of risk of bias on the results. Although a discussion on the impact of bias may be informative for the reader, it does not reflect a methodological aspect of meta-analyses. Fourthly, some comparisons between CBT vs any control group were only based on three RCTs, thereby limiting the study power. Lastly, recent literature reported weaknesses in the mainstream random-effects model (e.g. overreliance on normally distributed data, treating sample sizes, or weights as constant variables).^33^ We applied the random-effects model with restricted likelihood maximum for each analysis, potentially reducing the precision of our findings. We therefore advise future studies to keep weaknesses of random-effects models in mind. The main strength of our study, is that we are the first to demonstrate an overview of scientific findings in an area of inconsistent findings and created a hierarchy of evidence to inform clinicians.

In conclusion, we found suggestive evidence supporting the effectiveness of CBT in the treatment of delusions, hallucinations, general symptoms, and improvement of functioning measured directly after ending therapy in patients with SSD. We also found that CBT has limited or no effect on other clinically relevant outcomes and that evidence for effectiveness of CBT for general and positive symptoms may not be sustained at follow up. Research should focus on the etiology and pathophysiology of these outcomes and on evaluating other therapeutic interventions. Moreover, research should also evaluate how to sustain CBT improvements. For now, we propose that clinical guidelines concerning CBT for SSD should reconsider their recommendations.

## Contributors

SBn, JV, and LH conceived, designed the study and were responsible to write the protocol. JT and SBe primarily extracted the data and performed the statistical analysis. Any difference in opinion were resolved by discussion among SBn, JV, JT and SBe. SBe and JT had direct access to and verify the underlying study data. All authors (SBn, SBe, JT, JV, LH) contributed to the interpretation of the results, participated in manuscript writing, critically reviewed the manuscript, and approved the final manuscript for submission.

## Data sharing statement

All data in this review were from publicly available systematic reviews.

## Declaration of interests

All authors declare no competing interests.

## References

[1] Guideline N.C. (2014).

[2] (2013). American Psychiatric Association. http://www.dsm5.org.and/http://www.dsm5.org/Pages/Default.

[3] (2017). GGZ zorgstandaard psychose.

[4] Jauhar S. (2018). Cognitive behavioural therapy-a valid alternative to antipsychotics for psychosis?. Lancet Psychiatry.

[5] Jones C., Hacker D., Meaden A. (2018). Cognitive behavioural therapy plus standard care versus standard care plus other psychosocial treatments for people with schizophrenia. Cochrane Database Syst Rev.

[6] Wykes T., Steel C., Everitt B., Tarrier N. (2008). Cognitive behavior therapy for schizophrenia: effect sizes, clinical models, and methodological rigor. Schizophr Bull.

[7] Turner D.T., Burger S., Smit F., Valmaggia L.R., van der Gaag M. (2020). What constitutes sufficient evidence for case formulation-driven CBT for psychosis? Cumulative meta-analysis of the effect on hallucinations and delusions. Schizophr Bull.

[8] Turner D.T., van der Gaag M., Karyotaki E., Cuijpers P. (2014). Psychological interventions for psychosis: a meta-analysis of comparative outcome studies. Am J Psychiatry.

[9] McKenna P., Leucht S., Jauhar S., Laws K., Bighelli I. (2019). The controversy about cognitive behavioural therapy for schizophrenia. World Psychiatry.

[10] Jauhar S., McKenna P.J., Radua J., Fung E., Salvador R., Laws K.R. (2014). Cognitive-behavioural therapy for the symptoms of schizophrenia: systematic review and meta-analysis with examination of potential bias. Br J Psychiatry.

[11] Shea B.J., Grimshaw J.M., Wells G.A. (2007). Development of AMSTAR: a measurement tool to assess the methodological quality of systematic reviews. BMC Med Res Methodol.

[12] Fusar-Poli P., Radua J. (2018). Ten simple rules for conducting umbrella reviews. Evid Base Ment Health.

[13] Gosling C.J., Solanes A., Fusar-Poli P., Radua J. (2022). Metaumbrella: the first comprehensive suite to perform data analysis in umbrella reviews with stratification of the evidence. BMJ Ment Health.

[14] (2023). https://cran.r-project.org/web/packages/metaumbrella/metaumbrella.pdf.

[15] Langan D., Higgins J.P.T., Jackson D. (2019). A comparison of heterogeneity variance estimators in simulated random-effects meta-analyses. Res Synth Methods.

[16] Theodoratou E., Tzoulaki I., Zgaga L., Ioannidis J.P. (2014). Vitamin D and multiple health outcomes: umbrella review of systematic reviews and meta-analyses of observational studies and randomised trials. BMJ.

[17] Li X., Meng X., Timofeeva M. (2017). Serum uric acid levels and multiple health outcomes: umbrella review of evidence from observational studies, randomised controlled trials, and Mendelian randomisation studies. BMJ.

[18] Dragioti E., Karathanos V., Gerdle B., Evangelou E. (2017). Does psychotherapy work? An umbrella review of meta-analyses of randomised controlled trials. Acta Psychiatr Scand.

[19] Dragioti E., Solmi M., Favaro A. (2019). Association of antidepressant use with adverse health outcomes: a systematic umbrella review. JAMA Psychiatry.

[20] Bighelli I., Salanti G., Huhn M. (2018). Psychological interventions to reduce positive symptoms in schizophrenia: systematic review and network meta-analysis. World Psychiatry.

[21] Fusar-Poli P., Papanastasiou E., Stahl D. (2015). Treatments of negative symptoms in schizophrenia: meta-analysis of 168 randomised placebo-controlled trials. Schizophr Bull.

[22] Veerman S.R.T., Schulte P.F.J., de Haan L. (2017). Treatment for negative symptoms in schizophrenia: a comprehensive review. Drugs.

[23] Penney D., Sauvé G., Mendelson D., Thibaudeau É., Moritz S., Lepage M. (2022). Immediate and sustained outcomes and moderators associated with metacognitive training for psychosis: a systematic review and meta-analysis. JAMA Psychiatry.

[24] Vita A., Barlati S., Ceraso A. (2021). Effectiveness, core elements, and moderators of response of cognitive remediation for schizophrenia: a systematic review and meta-analysis of randomised clinical trials. JAMA Psychiatry.

[25] Thomas N. (2015). What's really wrong with cognitive behavioral therapy for psychosis?. Front Psychol.

[26] Leichsenring F., Steinert C., Rabung S., Ioannidis J.P.A. (2022). The efficacy of psychotherapies and pharmacotherapies for mental disorders in adults: an umbrella review and meta-analytic evaluation of recent meta-analyses. World Psychiatry.

[27] Solmi M., Croatto G., Piva G. (2022). Efficacy and acceptability of psychosocial interventions in schizophrenia: systematic overview and quality appraisal of the meta-analytic evidence. Mol Psychiatry.

[28] Gosling C.J., Cartigny A., Mellier B.C., Solanes A., Radua J., Delorme R. (2022). Efficacy of psychosocial interventions for Autism spectrum disorder: an umbrella review. Mol Psychiatry.

[29] Papatheodorou S.I., Evangelou E. (2022). Umbrella reviews: what they are and why we need them. Methods Mol Biol.

[30] Schlesinger S., Schwingshackl L., Neuenschwander M., Barbaresko J. (2019). A critical reflection on the grading of the certainty of evidence in umbrella reviews. Eur J Epidemiol.

[31] Gianfredi V., Nucci D., Amerio A., Signorelli C., Odone A., Dinu M. (2022). What can we expect from an umbrella review?. Adv Nutr.

[32] Training Cochrane https://training.cochrane.org/grade-approach.

[33] Shuster J.J. (2023). Meta-analysis of clinical trials in the 2020s and beyond: a paradigm shift needed. J Clin Transl Res.

