# Supplementary material for: Cognitive behavioural therapy for the treatment of schizophrenia spectrum disorders: an umbrella review of meta-analyses of randomised controlled trials
Source: eClinicalMedicine. 2024 Jan 5;67:102392. doi: 10.1016/j.eclinm.2023.102392 (PMC10809079; doi:10.1016/j.eclinm.2023.102392)
Supplement: Supplement [file mmc1.pdf]

Supplement

Content of supplementary material

Supplemental 1. Search strategy **1**

Supplemental 2. List of included meta-analyses **2**

Supplemental 3. List of meta-analyses providing sufficient data for statistical analysis **3**

Supplemental 4. Operationalization of each AMSTAR-2 items **4**

Supplemental Table 1. List of excluded studies, based on full-text evaluation **7**

Supplemental Table 2. Non-analyzable data primary outcome **11**

Supplemental Table 3. Non-analyzable data secondary outcome **15**

Supplemental Table 4. Sensitivity analysis **19**

Supplemental Table 5. The number of times randomized trials were included **21**

Supplemental figures 1-34. Forest plots for each outcome per follow-up assessment **27**

Supplement 1. Search strategy

Medline

(((meta-analysis[Title/Abstract])) AND ((schizo*) OR ("Schizophrenia Spectrum and Other Psychotic Disorders"[MeSH Terms]) OR (psychosis)) AND ((cogniti* AND (behavio* OR therap*)) OR (CBT)))

Embase

'meta analysis':ti,ab,kw AND ('schizophrenia spectrum disorder'/exp OR 'psychosis'/exp OR 'schizophrenia'/exp OR 'psycho') AND ('cognitive therapy' OR 'behavioral therapy' OR 'behavior therapy' OR 'cbt' OR 'behavioural therapy' OR 'behaviour therapy')

PsychInfo

AB (meta-analysis or meta analysis) AND TX (schizophrenia or psychosis or psychoses or psychotic disorder or schizophrenic disorder) AND TX (cbt or cognitive therapy or behavior therapy or behaviour therapy or behavioral therapy or cognitive behavior therapy)

Cochrane

MeSH descriptor: [Schizophrenia Spectrum and Other Psychotic Disorders] explode all trees AND (cogniti* NEXT (behavior* OR therap*)) (with filter only systematic reviews)

Supplement 2. List of included meta-analyses(1-26)

1. Jones C, Hacker D, Xia J, Meaden A, Irving CB, Zhao S, et al. Cognitive behavioural therapy plus standard care versus standard care for people with schizophrenia. Cochrane Database Syst Rev. 2018;12(12):Cd007964.
2. Jones C, Hacker D, Meaden A, Cormac I, Irving CB, Xia J, et al. Cognitive behavioural therapy plus standard care versus standard care plus other psychosocial treatments for people with schizophrenia. Cochrane Database Syst Rev. 2018;11(11):CD008712.
3. Barnicot K, Michael C, Trione E, Lang S, Saunders T, Sharp M, et al. Psychological interventions for acute psychiatric inpatients with schizophrenia-spectrum disorders: A systematic review and meta-analysis. Clin Psychol Rev. 2020;82:101929.
4. Turner DT, van der Gaag M, Karyotaki E, Cuijpers P. Psychological interventions for psychosis: a meta-analysis of comparative outcome studies. Am J Psychiatry. 2014;171(5):523-38.
5. Bighelli I, Rodolico A, García-Mieres H, Pitschel-Walz G, Hansen WP, Schneider-Thoma J, et al. Psychosocial and psychological interventions for relapse prevention in schizophrenia: a systematic review and network meta-analysis. Lancet Psychiatry. 2021;8(11):969-80.
6. Bighelli I, Salanti G, Huhn M, Schneider-Thoma J, Krause M, Reitmeir C, et al. Psychological interventions to reduce positive symptoms in schizophrenia: systematic review and network meta-analysis. World psychiatry : official journal of the World Psychiatric Association (WPA). 2018;17(3):316-29.
7. Burns AM, Erickson DH, Brenner CA. Cognitive-behavioral therapy for medication-resistant psychosis: a meta-analytic review. Psychiatric services (Washington, DC). 2014;65(7):874-80.
8. Jauhar S, McKenna PJ, Radua J, Fung E, Salvador R, Laws KR. Cognitive-behavioural therapy for the symptoms of schizophrenia: systematic review and meta-analysis with examination of potential bias. The British journal of psychiatry : the journal of mental science. 2014;204(1):20-9.
9. Kennedy L, Xyrichis A. Cognitive Behavioral Therapy Compared with Non-specialized Therapy for Alleviating the Effect of Auditory Hallucinations in People with Reoccurring Schizophrenia: A Systematic Review and Meta-analysis. Community Ment Health J. 2017;53(2):127-33.
10. Laws KR, Darlington N, Kondel TK, McKenna PJ, Jauhar S. Cognitive Behavioural Therapy for schizophrenia - outcomes for functioning, distress and quality of life: a meta-analysis. BMC Psychol. 2018;6(1):32.
11. Lincoln TM, Suttner C, Nestoriuc Y. Wirksamkeit kognitiver interventionen für schizophrenie. Psychologische Rundschau2008.
12. Lutgens D, Gariepy G, Malla A. Psychological and psychosocial interventions for negative symptoms in psychosis: systematic review and meta-analysis. The British journal of psychiatry : the journal of mental science. 2017;210(5):324-32.
13. Lynch D, Laws KR, McKenna PJ. Cognitive behavioural therapy for major psychiatric disorder: does it really work? A meta-analytical review of well-controlled trials. Psychological medicine. 2010;40(1):9-24.
14. Mc Glanaghy E, Turner D, Davis GA, Sharpe H, Dougall N, Morris P, et al. A network meta-analysis of psychological interventions for schizophrenia and psychosis: Impact on symptoms. Schizophrenia research. 2021;228:447-59.

15. Mehl S, Werner D, Lincoln TM. Does Cognitive Behavior Therapy for psychosis (CBTp) show a sustainable effect on delusions? A meta-analysis. Front Psychol. 2015;6:1450.
16. Naeem F, Khoury B, Munshi T, Ayub M, Lecomte T, Kingdon D, et al. Brief Cognitive Behavioral Therapy for Psychosis (CBTp) for Schizophrenia: Literature Review and Meta-analysis. International Journal of Cognitive Therapy2016.
17. Newton-Howes G, Wood R. Cognitive behavioural therapy and the psychopathology of schizophrenia: systematic review and meta-analysis. Psychol Psychother. 2013;86(2):127-38.
18. Pilling S, Bebbington P, Kuipers E, Garety P, Geddes J, Orbach G, et al. Psychological treatments in schizophrenia: I. Meta-analysis of family intervention and cognitive behaviour therapy. Psychological medicine. 2002;32(5):763-82.
19. Sarin F, Wallin L, Widerlöv B. Cognitive behavior therapy for schizophrenia: a meta-analytical review of randomized controlled trials. Nord J Psychiatry. 2011;65(3):162-74.
20. Todorovic A, Lal S, Dark F, De Monte V, Kisely S, Siskind D. CBTp for people with treatment refractory schizophrenia on clozapine: a systematic review and meta-analysis. J Ment Health. 2020:1-8.
21. Turner DT, Burger S, Smit F, Valmaggia LR, van der Gaag M. What Constitutes Sufficient Evidence for Case Formulation-Driven CBT for Psychosis? Cumulative Meta-analysis of the Effect on Hallucinations and Delusions. Schizophrenia bulletin. 2020;46(5):1072-85.
22. Turner DT, Reijnders M, van der Gaag M, Karyotaki E, Valmaggia LR, Moritz S, et al. Efficacy and Moderators of Cognitive Behavioural Therapy for Psychosis Versus Other Psychological Interventions: An Individual-Participant Data Meta-Analysis. Front Psychiatry. 2020;11:402.
23. Wykes T, Steel C, Everitt B, Tarrier N. Cognitive behavior therapy for schizophrenia: effect sizes, clinical models, and methodological rigor. Schizophrenia bulletin. 2008;34(3):523-37.
24. Velthorst E, Koeter M, van der Gaag M, Nieman DH, Fett AK, Smit F, et al. Adapted cognitive-behavioural therapy required for targeting negative symptoms in schizophrenia: meta-analysis and meta-regression. Psychological medicine. 2015;45(3):453-65.
25. van der Gaag M, Valmaggia LR, Smit F. The effects of individually tailored formulation-based cognitive behavioural therapy in auditory hallucinations and delusions: a meta-analysis. Schizophrenia research. 2014;156(1):30-7.
26. Bighelli IW, S., Reitmeir C, Schwermann F, Salahuddin N, Leucht S. Effects of psychological treatments on functioning in people with Schizophrenia: a systematic review and meta-analysis of randomized controlled trials. Eur Arch Psychiatry Clin Neurosci; 2023.

Supplemental 3. List of meta-analyses providing sufficient data for statistical analysis.

1. Jauhar S, McKenna PJ, Radua J, Fung E, Salvador R, Laws KR. Cognitive-behavioural therapy for the symptoms of schizophrenia: systematic review and meta-analysis with examination of potential bias. The British journal of psychiatry : the journal of mental science. 2014;204(1):20-9.
2. Jones C, Hacker D, Meaden A, Cormac I, Irving CB, Xia J, et al. Cognitive behavioural therapy plus standard care versus standard care plus other psychosocial treatments for people with schizophrenia. Cochrane Database Syst Rev. 2018;11(11):CD008712.
3. Jones C, Hacker D, Xia J, Meaden A, Irving CB, Zhao S, et al. Cognitive behavioural therapy plus standard care versus standard care for people with schizophrenia. Cochrane Database Syst Rev. 2018;12(12):Cd007964.
4. Todorovic A, Lal S, Dark F, De Monte V, Kisely S, Siskind D. CBTp for people with treatment refractory schizophrenia on clozapine: a systematic review and meta-analysis. J Ment Health. 2020:1-8.
5. Barnicot K, Michael C, Trione E, Lang S, Saunders T, Sharp M, et al. Psychological interventions for acute psychiatric inpatients with schizophrenia-spectrum disorders: A systematic review and meta-analysis. Clin Psychol Rev. 2020;82:101929.
6. Newton-Howes G, Wood R. Cognitive behavioural therapy and the psychopathology of schizophrenia: systematic review and meta-analysis. Psychol Psychother. 2013;86(2):127-38.
7. Lynch D, Laws KR, McKenna PJ. Cognitive behavioural therapy for major psychiatric disorder: does it really work? A meta-analytical review of well-controlled trials. Psychological medicine. 2010;40(1):9-24.
8. Bighelli I, Salanti G, Huhn M, Schneider-Thoma J, Krause M, Reitmeir C, et al. Psychological interventions to reduce positive symptoms in schizophrenia: systematic review and network meta-analysis. World psychiatry : official journal of the World Psychiatric Association (WPA). 2018;17(3):316-29.
9. Lutgens D, Gariepy G, Malla A. Psychological and psychosocial interventions for negative symptoms in psychosis: systematic review and meta-analysis. The British journal of psychiatry : the journal of mental science. 2017;210(5):324-32.
10. Turner DT, Reijnders M, van der Gaag M, Karyotaki E, Valmaggia LR, Moritz S, et al. Efficacy and

Moderators of Cognitive Behavioural Therapy for Psychosis Versus Other Psychological Interventions: An Individual-Participant Data Meta-Analysis. Front Psychiatry. 2020;11:402.

11. Velthorst E, Koeter M, van der Gaag M, Nieman DH, Fett AK, Smit F, et al. Adapted cognitive-behavioural therapy required for targeting negative symptoms in schizophrenia: meta-analysis and meta-regression. Psychological medicine. 2015;45(3):453-65.
12. Turner DT, Burger S, Smit F, Valmaggia LR, van der Gaag M. What Constitutes Sufficient Evidence for Case Formulation-Driven CBT for Psychosis? Cumulative Meta-analysis of the Effect on Hallucinations and Delusions. Schizophrenia bulletin. 2020;46(5):1072-85.
13. Mehl S, Werner D, Lincoln TM. Does Cognitive Behavior Therapy for psychosis (CBTp) show a sustainable effect on delusions? A meta-analysis. Front Psychol. 2015;6:1450.
14. van der Gaag M, Valmaggia LR, Smit F. The effects of individually tailored formulation-based cognitive behavioural therapy in auditory hallucinations and delusions: a meta-analysis. Schizophrenia research. 2014;156(1):30-7.
15. Laws KR, Darlington N, Kondel TK, McKenna PJ, Jauhar S. Cognitive Behavioural Therapy for schizophrenia - outcomes for functioning, distress and quality of life: a meta-analysis. BMC Psychol. 2018;6(1):32.
16. Bighelli IW, S., Reitmeir C, Schwermann F, Salahuddin N, Leucht S. Effects of psychological treatments on functioning in people with Schizophrenia: a systematic review and meta-analysis of randomized controlled trials. Eur Arch Psychiatry Clin Neurosci; 2023.

Supplemental 4. Operationalization of each AMSTAR-2 items, adopted from Shea et al. 2017.

Item 1: Did the research questions and inclusion criteria for the review include the components of Problem Intervention Control and Outcome (PICO)?

For Yes: appraisers should be confident that the four elements of PICO are described somewhere in the report.

For No: any element of the PICO is missing in the report.

Item 2: Did the report of the review contain an explicit statement that the review methods were established prior to conduct of the review and did the report justify any significant deviations from the protocol?

For Partial Yes: The authors state that they had a written protocol or guide that included all the following: review question(s), a search strategy, inclusion/exclusion criteria, a risk of bias assessment.

For Yes: authors should demonstrate that they have worked with a written protocol with independent verification (by a registration independent body, e.g. research ethics board or research office) before the review was undertaken. In addition, the protocol should be registered and should also have specified: a meta-analysis/synthesis plan, if appropriate, and, a plan for investigating causes of heterogeneity, justification for any deviations from the protocol.

For No: if any of the above mentioned elements are missing.

Item 3. Did the review authors explain their selection of the study designs for inclusion in the review?

For Yes: the review should satisfy one of the following elements: explanation for including only randomized controlled trial (RCTs).

For No: if the abovementioned elements are missing.

Item 4. Did the review authors use a comprehensive literature search strategy?

Partial Yes: all the following should be completed: searched at least 2 databases (relevant to research question), provided key word and/or search strategy, justified publication restrictions (e.g. language).

For Yes: in addition to all elements of partial yes, all the following has to be completed too: searched the reference lists / bibliographies of included studies. searched trial/study registries, included/consulted content experts in the field, if relevant, searched for grey literature, conducted search within 24 months of completion of the review

For No: the abovementioned is missing.

Item 5. Did the review authors perform study selection in duplicate?

For Yes: either one of the following: at least two reviewers independently agreed on selection of eligible studies and achieved consensus on which studies to include, or two reviewers selected a sample of eligible studies and achieved good agreement (at least 80 percent), with the remainder selected by one reviewer.

For No: the abovementioned is missing.

Item 6. Did the review authors perform data extraction in duplicate?

For Yes, either one of the following: at least two reviewers achieved consensus on which data to extract from included studies, or two reviewers extracted data from a sample of eligible studies and achieved good agreement (at least 80 percent), with the remainder extracted by one reviewer.

For No: the abovementioned elements are missing.

Item 7. Did the review authors provide a list of excluded studies and justify the exclusions?

For Partial Yes: provided a list of all potentially relevant studies that were read in full-text form but excluded from the review.

For Yes, must also have justified the exclusion from the review of each potentially relevant study.

For No: the abovementioned elements are missing.

Item 8. Did the review authors describe the included studies in adequate detail?

For Partial Yes (all the following): described populations, described interventions, described comparators, described outcomes, described research designs.

For Yes, should also have all the following: described population in detail, described intervention in detail (including doses if relevant), described comparator in detail (including doses if relevant), described study's setting, timeframe for follow-up.

For No: the abovementioned elements are missing.

Item 9. Did the review authors use a satisfactory technique for assessing the risk of bias (RoB) in individual studies that were included in the review?

For Partial Yes, must have assessed RoB from unconcealed allocation, and lack of blinding of patients and assessors when assessing outcomes (unnecessary for objective outcomes such as all-cause mortality).

For Yes, must also have assessed RoB from allocation sequence that was not truly random, and selection of the reported result from among multiple measurements or analyses of a specified outcome.

For No: the abovementioned elements are missing.

Item 10. Did the review authors report on the sources of funding for the studies included in the review?

For Yes: must have reported on the sources of funding for individual studies included in the review. Note: Reporting that the reviewers searched this information but it was not reported by study authors also qualifies.

For No: the abovementioned elements are missing.

Item 11. If meta-analysis was performed did the review authors use appropriate methods for statistical combination of results?

For Yes: the authors justified combining the data in a meta-analysis and, they used an appropriate weighted technique to combine study results and adjusted for heterogeneity if present. And investigated the causes of any heterogeneity.

For No: the abovementioned elements are missing.

Item 12. If meta-analysis was performed, did the review authors assess the potential impact of RoB in individual studies on the results of the meta-analysis or other evidence synthesis?

For Yes: included only low risk of bias RCTs or, if the pooled estimate was based on RCTs at variable RoB, the authors performed analyses to investigate possible impact of RoB on summary estimates of effect.

For no: the abovementioned elements are missing.

Item 13. Did the review authors account for RoB in individual studies when interpreting/ discussing the results of the review?

For Yes: included only low risk of bias RCTs or if RCTs with moderate or high RoB, and provided a discussion of the likely impact of RoB on the results.

For No: the abovementioned elements are missing.

Item 14. Did the review authors provide a satisfactory explanation for, and discussion of, any heterogeneity observed in the results of the review?

For Yes: there was no significant heterogeneity in the results or if heterogeneity was present the authors performed an investigation of sources of any heterogeneity in the results and discussed the impact of this on the results of the review.

For No: the abovementioned elements are missing.

Item 15. If they performed quantitative synthesis did the review authors carry out an adequate investigation of publication bias (small study bias) and discuss its likely impact on the results of the review?

For Yes: performed graphical or statistical tests for publication bias and discussed the likelihood and magnitude of impact of publication bias.

For No: the abovementioned elements are missing.

Or: no meta-analysis was performed.

Item 16. Did the review authors report any potential sources of conflict of interest, including any funding they received for conducting the review?

For Yes: the authors reported no competing interests or the authors described their funding sources and how they managed potential conflicts of interest.

For No: the abovementioned elements are missing.

Supplemental Table 1. List of excluded studies, based on full-text evaluation.

	Reason
Allott K, van-der-El K, Bryce S, Parrish EM, McGurk SR, Hetrick S, et al. Compensatory Interventions for Cognitive Impairments in Psychosis: A Systematic Review and Meta-Analysis. Schizophrenia bulletin. 2020;46(4):869-83.	Wrong intervention
Alvarez-Jiménez M, Parker AG, Hetrick SE, McGorry PD, Gleeson JF. Preventing the second episode: a systematic review and meta-analysis of psychosocial and pharmacological trials in first-episode psychosis. Schizophrenia bulletin. 2011;37(3):619-30.	Wrong intervention
Bighelli I, Huhn M, Schneider-Thoma J, Krause M, Reitmeir C, Wallis S, et al. Response rates in patients with schizophrenia and positive symptoms receiving cognitive behavioural therapy: a systematic review and single-group meta-analysis. BMC Psychiatry. 2018;18(1):380.	No meta-analysis of RCTs
Bighelli I, Leucht S. Psychological interventions for positive symptoms in schizophrenia: A network meta-analysis. Schizophr Bull. 2018;44:S305.	No meta-analysis of RCTs
Brand RM, McEnery C, Rossell S, Bendall S, Thomas N. Do trauma-focussed psychological interventions have an effect on psychotic symptoms? A systematic review and meta-analysis. Schizophr Res. 2018;195:13-22.	Wrong intervention
Broderick J, Knowles A, Chadwick J, Vancampfort D. Yoga versus standard care for schizophrenia. Cochrane Database of Systematic Reviews. 2015(10).	Wrong intervention
Burlingame GM, Svien H, Hoppe L, Hunt I, Rosendahl J. Group therapy for schizophrenia: A meta-analysis. Psychotherapy (Chic). 2020;57(2):219-36.	Wrong intervention
Burns T. Psychosocial interventions. CURR OPIN PSYCHIATRY. 1997;10(1):36-9.	No meta-analysis of RCTs
Cella M, Preti A, Edwards C, Dow T, Wykes T. Cognitive remediation for negative symptoms of schizophrenia: A network meta-analysis. Clinical psychology review. 2017;52:43-51.	Wrong intervention
d'Arma A, Isernia S, Di Tella S, Rovaris M, Valle A, Baglio F, et al. Social Cognition Training for Enhancing Affective and Cognitive Theory of Mind in Schizophrenia: A Systematic Review and a Meta-Analysis. J Psychol. 2021;155(1):26-58.	Wrong intervention
Dauwan M, Begemann MJ, Heringa SM, Sommer IE. Exercise Improves Clinical Symptoms, Quality of Life, Global Functioning, and Depression in Schizophrenia: A Systematic Review and Meta-analysis. Schizophrenia bulletin. 2016;42(3):588-99.	Wrong intervention
Dauwan M, Begemann MJH, Slot MIE, Lee EHM, Scheltens P, Sommer IEC. Physical exercise improves quality of life, depressive symptoms, and cognition across chronic brain disorders: a transdiagnostic systematic review and meta-analysis of randomized controlled trials. J Neurol. 2021;268(4):1222-46.	Wrong intervention
Dilk MN, Bond GR. Meta-analytic evaluation of skills training research for individuals with severe mental illness. J CONSULT CLIN PSYCHOL. 1996;64(6):1337-46.	Wrong diagnosis
Ducasse D, Fond G. [Acceptance and commitment therapy]. Encephale. 2015;41(1):1-9.	No meta-analysis of RCTs
Eichner C, Berna F. Acceptance and efficacy of Metacognitive Training (MCT) on positive symptoms and delusions in patients with schizophrenia: A meta-analysis taking into account important moderators. Schizophrenia Bulletin. 2016;42(4):952-62.	Wrong intervention
Firth J, Cotter J, Elliott R, French P, Yung AR. A systematic review and meta-analysis of exercise interventions in schizophrenia patients. Psychological medicine. 2015;45(7):1343-61.	Wrong intervention
Gottdiener WH. The benefits of individual psychotherapy for schizophrenic patients: A meta-analytic review of the psychotherapy outcome literature: ProQuest Information & Learning; 2001.	No meta-analysis of RCTs
Gray R, Bressington D, Ivanecka A, Hardy S, Jones M, Schulz M, et al. Is adherence therapy an effective adjunct treatment for patients with schizophrenia spectrum disorders? A systematic review and meta-analysis. BMC Psychiatry. 2016;16(1).	Wrong intervention

Grynszpan O, Perbal S, Pelissolo A, Fossati P, Jouvent R, Dubal S, et al. Efficacy and specificity of computer-assisted cognitive remediation in schizophrenia: A meta-analytical study. Psychological Medicine. 2011;41(1):163-73.	Wrong intervention
Hazell CM, Hayward M, Cavanagh K, Strauss C. A systematic review and meta-analysis of low intensity CBT for psychosis. Clin Psychol Rev. 2016;45:183-92.	No meta-analysis of RCTs
Kambeitz-Ilankovic L, Betz LT, Dominke C, Haas SS, Subramaniam K, Fisher M, et al. Multi-outcome meta-analysis (MOMA) of cognitive remediation in schizophrenia: Revisiting the relevance of human coaching and elucidating interplay between multiple outcomes. Neurosci Biobehav Rev. 2019;107:828-45.	Wrong intervention
Kingdon D, Turkington D. Cognitive therapy of psychosis: Research and implementation. Schizophr Res. 2019;203:62-5.	No meta-analysis of RCTs
Krabbendam L, Aleman A. Cognitive rehabilitation in schizophrenia: A quantitative analysis of controlled studies. Psychopharmacology (Berl). 2003;169(3):376-82.	Wrong intervention
Kurtz MM. Cognitive remediation for schizophrenia: Current status, biological correlates and predictors of response. Expert Rev Neurother. 2012;12(7):813-21.	Wrong intervention
Kurtz MM, Richardson CL. Social cognitive training for schizophrenia: a meta-analytic investigation of controlled research. Schizophrenia bulletin. 2012;38(5):1092-104.	Wrong intervention
Leichsenring F, Rabung S. Effectiveness of long-term psychodynamic psychotherapy: A meta-analysis. J Am Med Assoc. 2008;300(13):1551-65.	Wrong diagnosis
Lejeune JA, Northrop A, Kurtz MM. A Meta-analysis of Cognitive Remediation for Schizophrenia: Efficacy and the Role of Participant and Treatment Factors. Schizophrenia bulletin. 2021;47(4):997-1006.	Wrong intervention
Liu YC, Tang CC, Hung TT, Tsai PC, Lin MF. The Efficacy of Metacognitive Training for Delusions in Patients With Schizophrenia: A Meta-Analysis of Randomized Controlled Trials Informs Evidence-Based Practice. Worldviews Evid Based Nurs. 2018;15(2):130-9.	Wrong intervention
Lopez-Morinigo J-D, Ajnakina O, Martínez AS-E, Escobedo-Aedo P-J, Ruiz-Ruano VG, Sánchez-Alonso S, et al. Can metacognitive interventions improve insight in schizophrenia spectrum disorders? A systematic review and meta-analysis. Psychological Medicine. 2020;50(14):2289-301.	Wrong intervention
McGurk SR, Twamley EW, Sitzer DI, McHugo GJ, Mueser KT. A meta-analysis of cognitive remediation in schizophrenia. The American Journal of Psychiatry. 2007;164(12):1791-802.	wrong intervention
Naeem F, Farooq S, Kingdon D. Cognitive behavioural therapy (brief versus standard duration) for schizophrenia. Cochrane Database of Systematic Reviews. 2015(10).	No meta-analysis of RCTs
Penney D, Sauvé G, Mendelson D, Thibaudeau É, Moritz S, Lepage M. Immediate and Sustained Outcomes and Moderators Associated With Metacognitive Training for Psychosis: A Systematic Review and Meta-analysis. JAMA Psychiatry. 2022;79(5):417-29.	No meta-analysis of RCTs
Pilling S, Bebbington P, Kuipers E, Garety P, Geddes J, Martindale B, et al. Psychological treatments in schizophrenia: II Meta-analyses of randomized controlled trials of social skills training and cognitive remediation. Psychological Medicine. 2002;32(5):783-91.	Wrong intervention
Prikken M, Konings MJ, Lei WU, Begemann MJH, Sommer IEC. The efficacy of computerized cognitive drill and practice training for patients with a schizophrenia-spectrum disorder: A meta-analysis. Schizophrenia research. 2019;204:368-74.	Wrong intervention
Riehle M, Pillny M, Buggisch S, Lincoln T. The efficacy of psychosocial treatments for patients with present negative symptoms: A meta-analysis. Eur Arch Psychiatry Clin Neurosci. 2017;267(1):S52.	Wrong intervention
Roelofs RL, Wingbermühle E, Egger JIM, Kessels RPC. Social Cognitive Interventions in Neuropsychiatric Patients: A Meta-Analysis. Brain Impairment. 2017;18(1):138-73.	Wrong intervention
Sauvé G, Lavigne KM, Pochiet G, Brodeur MB, Lepage M. Efficacy of psychological interventions targeting cognitive biases in schizophrenia: A systematic review and meta-analysis. Clinical psychology review. 2020;78:101854.	Wrong intervention

Schwalbe C, Gearing R. The moderating effect of adherence-promoting interventions with clients on evidence-based practices for children and adolescents with mental health problems. Am J Orthopsychiatry. 2012;82(1):146-55.	Wrong diagnosis
Scott AJ, Webb TL, Rowse G. Self-help interventions for psychosis: A meta-analysis. Clin Psychol Rev. 2015;39:96-112.	Wrong intervention
Stafford MR, Mayo-Wilson E, Loucas CE, James A, Hollis C, Birchwood M, et al. Efficacy and safety of pharmacological and psychological interventions for the treatment of psychosis and schizophrenia in children, adolescents and young adults: a systematic review and meta-analysis. PloS one. 2015;10(2):e0117166.	Less than 2 RCTs in meta-analysis
Turner DT, McGlanaghy E, Cuijpers P, van der Gaag M, Karyotaki E, MacBeth A. A Meta-Analysis of Social Skills Training and Related Interventions for Psychosis. Schizophrenia bulletin. 2018;44(3):475-91.	Wrong intervention
Turner WA, Casey LM. Outcomes associated with virtual reality in psychological interventions: where are we now? Clin Psychol Rev. 2014;34(6):634-44.	Wrong intervention
Valiente C, Espinosa R, Trucharte A, Nieto J, Martínez-Prado L. The challenge of well-being and quality of life: A meta-analysis of psychological interventions in schizophrenia. Schizophr Res. 2019;208:16-24.	Wrong intervention
Vita A, Barlati S, Ceraso A, Nibbio G, Ariu C, Deste G, et al. Effectiveness, core elements, and moderators of response of cognitive remediation for schizophrenia: A systematic review and meta-analysis of randomized clinical trials. JAMA Psychiatry. 2021;78(8):848-58.	Wrong intervention
Wang W, Zhou Y, Chai N, Liu D. Cognitive-behavioural therapy for personal recovery of patients with schizophrenia: A systematic review and meta-analysis. Gen Psychiatr. 2019;32(4):e100040.	Retracted
Wood L, Williams C, Billings J, Johnson S. A systematic review and meta-analysis of cognitive behavioural informed psychological interventions for psychiatric inpatients with psychosis. Schizophrenia research. 2020;222:133-44.	Wrong intervention, CBT was in some studies only a subcomponent
Wykes T, Huddy V, Cellard C, McGurk SR, Czobor P. A meta-analysis of cognitive remediation for schizophrenia: Methodology and effect sizes. Am J Psychiatry. 2011;168(5):472-85.	Wrong intervention
Yeo H, Yoon S, Lee J, Kurtz MM, Choi K. A meta-analysis of the effects of social-cognitive training in schizophrenia: The role of treatment characteristics and study quality. Br J Clin Psychol. 2022;61(1):37-57.	Wrong intervention
Zimmermann G, Favrod J, Trieu VH, Pomini V. The effect of cognitive behavioral treatment on the positive symptoms of schizophrenia spectrum disorders: A meta-analysis. Schizophrenia Research. 2005;77(1):1-9.	No meta-analysis of RCTs

Supplemental Table 2. Non-analyzable data ranked by significance value and publication year.

Outcome	First authors and year		Intervention and control group	RCTs	Follow-up	Sample size		Effect size	Type of effect size	P-value
General psychopathology	McGlanaghy et al. 2021		CBT vs TAU1 (network)	-	EoT	-		0·46 (-0·67; 0·24)	SMD	sig.
			CBT vs TAU2 (network)	-	EoT	-		-0·32 (-0·80;-0·16)	SMD	sig.
			CBT vs MPE (network)	-	EoT	-		-0·53 (-0·91· -0·15)	SMD	sig.
			CBT vs PE (network)	-	EoT	-		-0·24 (-0·48-0)	SMD	sig.
			CBT vs BF (network)	-	EoT	-		-0·33 (-0·63;-0·03)	SMD	sig.
	Bighelli et al. 2021		CBT vs FP (network)	-	EoT	-		-0·46 (-0·83; -0·09)	SMD	sig.
			CBT vs TAU (pairways)	6	EoT	-		-0·41 (-0·67; -0·16)	SMD	sig.
			CBT vs MIF (network)	-	EoT	-		-0·69 (-1·31; -0·07)	SMD	sig.
	Bighelli et al. 2018		CBT vs TAU (network)	-	EoT	-		-0·38 (-0·56; -0·2)	SMD	sig.
			CBT vs TAU (pairways)	15	EoT	1208		-0·36 (-0·54; -0·17)	SMD	sig.
			CBT vs WL (pairways)	3	EoT	147		-0·50 (-0·96; -0·05)	SMD	sig.
	Naeem et al 2016		B-CBT vs TAU	6	EoT	-		0·43 (0·24-0·63)	Hedges' G	<0.0001
			B-CBT vs TAU	4	1-18 months	-		0·38 (0·17-0·59)	Hedges' G	<0.0001
			B-CBT vs mixed	8	EoT	-		0·38 (0·2-0·57)	Hedges' G	<0.0001
			B-CBT vs mixed	5	1-18 months	-		0·31 (0·13-0·5)	Hedges' G	<0.05
	Turner, van der Gaag et al 2014		CBT vs OPT	22	EoT	-		0·16 (0·04-0·28)	Hedges' G	<0.05
			CBT vs befriending	6	EoT	-		0·42 (0·15; 0·69)	Hedges' G	<0.05
	Sarin et al. 2011		CBT vs OPT (PANSS total)	5	3-15 months	359		-4·8 (-8·01·-1·59)	MD	3.0 x10^{-3}
			CBT vs TAU (PANSS total)	4	3-15 months	452		-2·87 (-5·67·-0·08)	MD	0,04
			CBT vs OPT (BPRS, CPS, PANS-g)	8	3-15 months	537		-0·21 (-0·38· -0·04)	SMD	0,02
	Lincoln et al. 2008		CBT vs TAU	6	9-28	663		0·35 (0·21-0·48)	SMD	0.00
			CBT vs TAU	9	EoT	908		0·25 (0·14 -0·36)	SMD	0.00
			CBT vs AT	6	9-28	416		0·24 (0·07-0·42)	SMD	0.01
	Pilling et al. 2002		CBT vs TAU	4	EoT	273		0·28 (0·15; 0·51)	OR	sig.
			CBT vs TAU	4	EoT	273		0·28 (0·15; 0·51)	OR	sig.
	McGlanaghy et al. 2021 (network meta-analysis)		CBT vs TAU0	-	EoT	-		-0·17 (-0·87;0·53)	SMD	NS
			CBT vs MCT	-	EoT	-		0·01 (-0·78 – 0·8)	SMD	NS
			CBT vs SST	-	EoT	-		0·26 (-0·56· 1·07)	SMD	NS
			CBT vs CRSS	-	EoT	-		-0·07 (-0·39· 0·25)	SMD	NS
			CBT vs EMDR	-	EoT	-		-0·08 (-0·89· 0·73)	SMD	NS
			CBT vs FT	-	EoT	-		-0·03 (-0·37· 0·32)	SMD	NS
			CBT vs HIT	-	EoT	-		-0·32 (-1·03· 0·39)	SMD	NS
			CBT vs MCT	-	EoT	-		0·07 (-0·43· 0·58)	SMD	NS
			CBT vs all	-	EoT	-		-0·13 (-1 - 075)	SMD	NS
			CBT vs CR & MCT	-	EoT	-		0·1 (-0·16-0·36)	SMD	NS
			CBT vs SST	-	EoT	-		0 (-0·25· 0·24)	SMD	NS
			CBT vs TAU	-	EoT	-		0·42 (-0·14· 0·97)	SMD	NS
			CBT vs WB	-	EoT	-		-0·13 (-0·81· 0·55)	SMD	NS
			CBT vs OT	-	EoT	-		0·18 (-0·27; 0·63)	SMD	NS

			CBT vs SC	-	EoT	-		-0·13 (-0·37; 0·1)	SMD	NS
	Bighelli et al. 2021		CBT vs CM (network)	-	EoT	-		-0·4 (-0·8; 0·01)	SMD	NS
			CBT vs CT (network)	-	EoT	-		-0·32 (-0·97; 0·34)	SMD	NS
			CBT vs FI (network)	-	EoT	-		-0·08 (-0·44; 0·28)	SMD	NS
			CBT vs FS (network)	-	EoT	-		-0·45 (-1·18; 0·28)	SMD	NS
			CBT vs II (network)	-	EoT	-		-0·01 (-0·38; 0·36)	SMD	NS
			CBT vs ST (network)	-	EoT	-		-0·18 (-0·69; 0·32)	SMD	NS
			CBT vs TAU (network)	-	EoT	-		-0·38 (-0·62; -0·15)	SMD	NS
			CBT vs TI (network)	-	EoT	-		-0·13 (-0·97; 0·71)	SMD	NS
			CBT vs MI (network)	-	EoT	-		-0·09 (-0·7; 0·53)	SMD	NS
			CBT vs PE (network)	-	EoT	-		-0·08 (-0·43; 0·28)	SMD	NS
			CBT vs HE (network)	-	EoT	-		-0·19 (-1·09; 0·7)	SMD	NS
			CBT vs ACT (network)	-	EoT	-		-0·17 (-0·6; 0·26)	SMD	NS
	Bighelli et al. 2018		CBT vs HFIT (network)	-	EoT			-0·26 (-0·98; 0·46)	SMD	NS
			CBT vs FI (network)	-	EoT			-0·28 (-0·94; 0·39)	SMD	NS
			CBT vs SST (network)	-	EoT			-0·16 (-0·75; 0·43)	SMD	NS
			CBT vs ST (network)	-	EoT			-0·33 (-0·86; 0·2)	SMD	NS
			CBT vs IC (network)	-	EoT			-0·17 (-0·46; 0·11)	SMD	NS
			CBT vs WL (network)	-	EoT			-0·36 (-0·80; 0·07)	SMD	NS
			CBT vs ACT (network)	-	EoT			-0·33 (-1·03; 0·37)	SMD	NS
			CBT vs EFC (network)	-	EoT			0·16 (-1·13; 1·45)	SMD	NS
			CBT vs IC (pairways)	7	EoT	492		-0·17 (-0·46; 0·11)	SMD	NS
			CBT vs MT (network)	-	EoT			-0·49 (-1·26; 0·28)	SMD	NS
	Turner, van der Gaag et al 2014		CBT vs OPT (PANSS)	8	EoT	-		0·1 (-0·13; 0·32)	Hedges' G	NS
			CBT vs supportive counseling	8	EoT	-		0·1 (-0·1; 0·3)	Hedges' G	NS
	Burns et al. 2014		CBT vs mixed (therapy resistance)	12	EoT	639 (CBT + controls)		0·52 (0·35 -0·7)	Hedges' G	NS
			CBT vs mixed (therapy resistance)	7	3-8 months	381 (CBT + controls)		0·4 (0·2-0·6)	Hedges' G	NS
	Sarin et al. 2011		CBT vs OPT (PANSS total)	5	EoT	357		-2·09 (-4·96-0·78)	MD	0,15
			CBT vs TAU (PANSS total)	4	EoT	529		-1·25 (-3·75- 1·25)	MD	0,33
			CBT vs OPT (BPRS, CPS, PANS-g)	9	EoT	571		-0·07 (-0·23-0·1)	SMD	0,43
			CBT vs TAU (BPRS, CPS, PANS-g)	4	EoT	476		-0·1 (-0·29-0·08)	SMD	0,26
			CBT vs TAU (BPRS, CPS, PANS-g)	4	3-15 months	469		-0·14 (-0·32· 0·05)	SMD	0,14
	Lincoln et al. 2008		CBT vs AT	10	EoT	559		0·07 (-0·22 -0·36)	SMD	0,64
Positive symptoms	Bighelli et al. 2021		CBT vs ACT (network)	-	EoT	-		-0·64 (-1·25; -0·02)	SMD	sig.
			CBT vs TAU (pairways)	6	EoT	-		-0·27 (-0·43; -0·1)	SMD	sig.
	Jones, Hacker, Xia et al. 2018		CBT vs TAU	11	< 6 months	-		-3·11 (-4·97; -1·24)	MD	sig.
			CBT vs TAU	11	6-12 months	-		-1·23 (-1·9 ;-0·55)	MD	sig.

	Bighelli et al. 2018		CBT vs TAU (pairways)	18	EoT			-0·28 (-0·44; -0·12)	SMD	sig.
			CBT vs IC (network)	-	EoT	-		-0·29 (-0·55; -0·03)	SMD	sig.
			CBT vs IC (pairways)	7	EoT			-0·34 (-0·60;-0·07)	SMD	sig.
	Burns et al. 2014		CBT vs mixed (therapy resistance)	7	3-8 months	365 (CBT + controls)		0·41 (0·2-0·61)	Hedges' G	0.05
	Turner, van der Gaag et al. 2014		CBT vs OPT	17	EoT	-		0·16 (0·04;0·28)	Hedges' G	<0.05
			CBT vs supportive counseling	6	EoT	-		0·23 (0·01;0·44)	Hedges' G	<0.05
	Sarin et al. 2011		CBT vs OPT	7	3-15 months	483		-1·01 (-1·85·-0·18)	MD	0,02
	Lincoln et al. 2008		CBT vs TAU	6	EoT	493		0·21 (0·04-0·39)		0.02
	Wykes et al. 2008		CBT vs Mixed	32	EoT	1918		0·372 (0·228-0·516)	SMD	sig.
	Bighelli et al. 2021		CBT vs CM (network)	-	EoT	-		-0·15 (-0·46; 0·16)	SMD	NS
			CBT vs FI (network)	-	EoT	-		-0·1 (-0·41; 0·21)	SMD	NS
			CBT vs II (network)	-	EoT	-		-0·02 (-0·33; 0·29)	SMD	NS
			CBT vs RG (network)	-	EoT	-		-0·07 (-0·54; 0·4)	SMD	NS
			CBT vs TAU (network)	-	EoT	-		-0·29 (-0·45; -0·13)	SMD	NS
			CBT vs FP (network)	-	EoT	-		-0·02 (-0·28; 0·23)	SMD	NS
			CBT vs PE (network)	-	EoT	-		-0·13 (-0·49; 0·22)	SMD	NS
			CBT vs HE (network)	-	EoT	-		-0·2 (-0·85; 0·44)	SMD	NS
			CBT vs MI (network)	-	EoT	-		-0·17 (-0·58; 0·24)	SMD	NS
			CBT vs TI (network)	-	EoT	-		-0·51 (-1·24; 0·22)	SMD	NS
	Bighelli et al. 2018		CBT vs ACT (network)	-	EoT	-		-0·52 (-1·17; 0·14)	SMD	NS
			CBT vs AVATAR (network)	-	EoT	-		-0·09 (-0·71; 0·54)	SMD	NS
			CBT vs EFC (network)	-	EoT	-		-0·06 (-1·31; 1·19)	SMD	NS
			CBT vs FI (network)	-	EoT	-		-0·15 (-0·79; 0·48)	SMD	NS
			CBT vs HFIT (network)	-	EoT	-		-0·40 (-1·07; -0·28)	SMD	NS
			CBT vs MF (network)	-	EoT	-		-0·17 (-0·71; 0·38)	SMD	NS
			CBT vs MT (network)	-	EoT	-		-0·07 (-0·44; 0·30)	SMD	NS
			CBT vs SST(network)	-	EoT	-		-0·21 (-0·75; 0·34)	SMD	NS
	Burns et al. 2014		CBT vs mixed (therapy resistance)	9	EoT	465 (CBT + controls)		0·47 (0·27- 0·67)	Hedges' G	NS
	Sarin et al. 2011		CBT vs OPT	7	EoT	504		-0·55 (-1·37-0·27)	MD	0,19
			CBT vs TAU	3	EoT	417		-0·62 (-1·86-0·61)	MD	0,32
			CBT vs TAU	3	3-15 months	415		-0·67 (-1·7-0·36)	MD	0,20
	Lincoln et al. 2008		CBT vs AT	8	EoT	445		0·24 (-0·2 -0·68)	SMD	0,29
			CBT vs AT	6	9-28 months	338		0·16 (0·01 -0·32)	SMD	0,06
			CBT vs TAU	4	9-28 months	663		0·2 (-0·05-0·44)	SMD	0,12
Negative symptoms	Bighelli et al. 2021		CBT vs CM (network)	-	EoT	-		-0·37 (-0·58; -0·16)	SMD	sig.
			CBT vs TAU (pairways)	6	EoT	-		-0·3 (-0·47; -0·14)	SMD	sig.
			CBT vs TI (network)	-	EoT	-		-0·76 (-1·47; -0·04)	SMD	sig.
	Jones, Hacker, Xia et al. 2018		CBT vs TAU	12	< 6 months	-		-3·35 (-3·84; -2·85)	MD	1.0×10^{-5}

			CBT vs TAU	13	6-12 months	-		-1·43 (-1·94 ; -0·93)	MD	1.0 x10^{-5}
			CBT vs TAU	13	>12 months	-		-1·47 (-1·94 ;-0·99)	MD	1.0 x10^{-4}
	Bighelli et al. 2018		CBT vs TAU (network)	-	EoT	-		-0·16 (-0·29; -0·03)	SMD	sig.
			CBT vs TAU (pairways)	11	EoT	916		-0·15 (-0·29; -0·02)	SMD	sig.
	Lincoln et al. 2008		CBT vs mixed	8	EoT	236		0·56 (0·25-0·86)	SMD	-
	Wykes et al. 2008		CBT vs mixed	23	EoT	1268		0·44 (0·17; -0·70)	SMD	sig.
	Bighelli et al. 2021		CBT vs FI (network)	-	EoT	-		-0·34 (-0·69; 0·01)		NS
			CBT vs FP (network)	-	EoT	-		-0·17 (-0·44; 0·11)		NS
			CBT vs ST (network)	-	EoT	-		-0·18 (-0·46; 0·1)		NS
			CBT vs TAU (network)	-	EoT	-		-0·31 (-0·47; -0·15)		NS
			CBT vs HE (network)	-	EoT	-		-0·08 (-0·72; 0·57)		NS
			CBT vs MI (network)	-	EoT	-		-0·14 (-0·55; 0·27)		NS
			CBT vs ACT (network)	-	EoT	-		-0·19 (-0·78; 0·41)		NS
			CBT vs II (network)	-	EoT	-		-0·17 (-0·47; 0·13)		NS
			CBT vs PE (network)	-	EoT	-		-0·23 (-0·58; 0·11)		NS
	Bighelli et al. 2018		ACT (network)	-	EoT	-		0·05 (-0·40; 0·49)	SMD	NS
			AVATAR (network)	-	EoT	-		-0·14 (-0·55; 0·26)	SMD	NS
			FI (network)	-	EoT	-		-0·18 (-0·69; 0·33)	SMD	NS
			HFIT (network)	-	EoT	-		-0·09 (-0·59; 0·41)	SMD	NS
			IC (network)	-	EoT	-		-0·09 (-0·26; 0·08)	SMD	NS
			MT (network)	-	EoT	-		-0·16 (-0·80; 0·48)	SMD	NS
			SST (network)	-	EoT	-		0·01 (-0·37; 0·39)	SMD	NS
			ST (network)	-	EoT	-		0·00 (-0·65; 0·65)	SMD	NS
			CBT vs WL (network)	-	EoT	-		-0·12 (-0·48; 0·24)	SMD	NS
			CBT vs IC (pairways)	8	EoT	558		-0·09 (-0·26; 0·08)	SMD	NS
			CBT vs FI (pairways)	-	EoT	-		-0·19 (-0·78; 0·4)		NS
	Turner, van der Gaag et al. 2014		CBT vs OPT	15	EoT	-		0·04 (-0·09; 0·16)	Hedges' G	NS
	Sarin et al. 2011		CBT vs OPT	8	3-15 months	452		-0·2 (-0·39;-0·02)	SMD	0,03
	Sarin et al. 2011		CBT vs OPT	8	EoT	461		-0·1 (-0·28- 0·09)	SMD	0,31
			CBT vs TAU	3	EoT	328		-0·10 (-0·32; 0·11)	SMD	0,35
			CBT vs TAU	3	3-15 months	318		-0·21 (-0·43; 0·01)	SMD	0,07
Delusions	Turner, Burger et al 2020		CBT vs TAU	22	EoT	-		0·36 (0·2 ; 0·52)	Hedges' G	<0.01
	Naeem et al. 2016		B-CBT vs TAU	4	EoT	-		0·56 (0·09-1·03)	Hedges' G	<0.05
	Turner, Burger et al 2020		CBT vs AT	7	EoT	-		0·23 (-0·19; 0·55)	Hedges' G	NS
Hallucinations	Turner, Burger et al. 2020		CBT vs AT	8	EoT	-		0·34 (0·15 ; 0·53)	Hedges' G	<0.01
			CBT vs TAU	22	EoT	-		0·35 (0·18 ; 0·52)	Hedges' G	<0.01
	Naeem et al. 2016		B-CBT vs TAU	3				0·45 (0·18-0·72)	Hedges' G	5.0 x10^{-3}
	Sarin et al. 2011		CBT vs OPT	5	EoT	268		-1·04 (3·41-1·33)	MD	0,39
			CBT vs OPT	4	3-15 months	239		-1·58 –(4·23·-1·07)	MD	0,24

Supplemental Table 3. Non-analyzable data of secondary outcomes, ranked by significance value and publication year.

Outcome	First author(s) and year		Intervention and control group	RCTs	Follow-up	Cases		Effect size	Type of effect size	P-value
Affective symptoms	Jones, Hacker, Xia et al. 2018		CBT vs TAU	-	< 6 months	-		-4·86 (-5·75 ; -3·96)	MD	1.0×10^{-4}
			CBT vs TAU	-	>12 months	-		-1.00 (-1·82 ; -0·18)	MD	0,02
			CBT vs TAU	-	6-12 months	-		-0·80 (-1·7 ; 0·09)	MD	0,08
Anxiety	-									
Depressive symptoms	Bighelli et al. 2021		CBT vs CM (network)	-	EoT	-		-0·12 (-2·24; 2·01)	SMD	NS
			CBT vs ACT (network)	-	EoT	-		0 (-2·2; 2·19)	SMD	NS
			CBT vs TI (network)	-	EoT	-		-0·06 (-2·29; 2·17)	SMD	NS
			CBT vs FI (network)	-	EoT	-		-0·39 (-1·85; 1·08)	SMD	NS
			CBT vs TAU (pairways)	3	EoT	-		0·15 (-0·98; 1·27)	SMD	NS
			CBT vs II (network)	-	EoT	-		-0·04 (-2·2; 2·11)	SMD	NS
			CBT vs PE (network)	-	EoT	-		-0·07 (-2·23; 2·1)	SMD	NS
			CBT vs TAU (network)	-	EoT	-		-0·04 (-1·14; 1·05)	SMD	NS
	Bighelli et al. 2018		CBT vs HFIT (network)	-	EoT	-		-0·31 (-0·86; 0·25)	SMD	NS
			CBT vs MF (network)	-	EoT	-		-0·23 (-0·73; 0·27)	SMD	NS
			CBT vs AVATAR (network)	-	EoT	-		-0·19 (-0·73 ;0·35)	SMD	NS
			CBT vs EFC (network)	-	EoT	-		-0·25 (-1·45; 0·95)	SMD	NS
			CBT vs FI (network)	-	EoT	-		-0·48 (-1·04; 0·08)	SMD	NS
			CBT vs IC (network)	-	EoT	-		-0·20 (-0·53; 0·14)	SMD	NS
			CBT vs TAU (network)	-	EoT	-		-0·14 (-0·32; 0·03)	SMD	NS
			CBT vs WL (network)	-	EoT	-		-0·05 (-0·43; 0·33)	SMD	NS
			CBT vs IC (pair)	3	EoT	183		-0·20 (-0·53; 0·14)	SMD	NS
			CBT vs TAU (pair)	11	EoT	697		-0·14 (-0·32; 0·03)	SMD	NS
	Sarin et al. 2011		CBT vs OPT	3	EoT	150		-1·03 (-4·31; 2·24)	MD	0,54
			CBT vs TAU	3	EoT	356		0·92 (-1·78; 3·61)	MD	0,51
			CBT vs OPT	3	3-15	139		-3·25 (-6·77; 0·27)	MD	0,07
	Lincoln et al. 2008		CBT vs AT	3	EoT	295		0·52 (0·14; 0·91)	-	-
Rehospitalisation	Lincoln et al. 2008		CBT vs AT	4	-	319		0·01 (-0·21 ; 0·24)	-	NS
			CBT vs TAU	6	-	906		0·26 (0·13; 0·39)	-	sig.
Relapse/rehospitalisation	Bighelli et al. 2021		CBT vs CM (network)	-	6-12 months	-		0·50 (0·26; 0·96)	OR	sig.
			CBT vs FS (network)	-	6-12 months	-		0·21 (0·07; 0·64)	OR	sig.
			CBT vs FP (network)	-	>12 months	-		0·41 (0·17; 0·97)	OR	sig.
			CBT vs FI (network)	-	>12 months	-		0·38 (0·19; 0·77)	OR	sig.
			CBT vs RPP (network)	-	>12 months	-		0·21 (0·05; 0·98)	OR	sig.
			CBT vs RG (network)	-	<6 months	-		0·29 (0·09; 0·97)	OR	sig.
			CBT vs TAU (pairways)	4	6-12 months	-		0·45 (0·26; 0·79)	OR	sig.
	Bighelli et al. 2021		CBT vs ACT (network)	-	6-12 months			0·66 (0·3; 1·46)	OR	NS
			CBT vs ACTP (network)	-	6-12 months			1·15 (0·32; 4·12)	OR	NS
			CBT vs FP(network)	-	6-12 months			0·8 (0·43; 1·5)	OR	NS
			CBT vs HE(network)	-	6-12 months			0·2 (0·03; 1·31)	OR	NS

			CBT vs II (network)	-	6-12 months			0·73 (0·4; 1·34)	OR	NS
			CBT vs MI (network)	-	6-12 months			0·34 (0·04; 2·85)	OR	NS
			CBT vs PE (network)	-	6-12 months			0·72 (0·38; 1·36)	OR	NS
			CBT vs RE (network)	-	6-12 months			0·64 (0·34; 1·2)	OR	NS
			CBT vs RG (network)	-	6-12 months			0·57 (0·22; 1·43)	OR	NS
			CBT vs SST (network)	-	6-12 months			0·73 (0·35; 1·52)	OR	NS
			CBT vs ST (network)	-	6-12 months			0·85 (0·42; 1·7)	OR	NS
			CBT vs TAU (network)	-	6-12 months			0·45 (0·27; 0·75)	OR	NS
			CBT vs TI (network)	-	6-12 months			0·71 (0·28; 1·78)	OR	NS
			CBT vs CT (network)	-	6-12 months			0·69 (0·14; 3·36)	OR	NS
			CBT vs RPP (network)	-	6-12 months			0·73 (0·27; 2·01)	OR	NS
			CBT vs FI (network)	-	6-12 months			0·77 (0·42; 1·43)	OR	NS
			CBT vs CM (network)	-	<6 months			0·77 (0·30; 1·95)	OR	NS
			CBT vs CT (network)	-	<6 months			0·92 (0·12; 6·82)	OR	NS
			CBT vs FI (network)	-	<6 months			0·90 (0·37; 2·19)	OR	NS
			CBT vs FP (network)	-	<6 months			0·84 (0·34; 2·07)	OR	NS
			CBT vs FS (network)	-	<6 months			0·41 (0·11; 1·61)	OR	NS
			CBT vs PE (network)	-	<6 months			0·82 (0·35; 1·89)	OR	NS
			CBT vs SST (network)	-	<6 months			0·16 (0·02; 1·54)	OR	NS
			CBT vs RE (network)	-	<6 months			0·93 (0·39; 2·18)	OR	NS
			CBT vs TI (network)	-	<6 months			0·72 (0·17; 3·1)	OR	NS
			CBT vs II (network)	-	<6 months			0·79 (0·34; 1·81)	OR	NS
			CBT vs ACTP (network)	-	<6 months			0·69 (0·19; 2·57)	OR	NS
			CBT vs RPP (network)	-	<6 months			0·71 (0·22; 2·28)	OR	NS
			CBT vs ACT (network)	-	<6 months			0·36 (0·09; 1·37)	OR	NS
			CBT vs TAU (network)	-	<6 months			0·57 (0·27; 1·19)	OR	NS
			CBT vs TAU (network)	-	>12 months			1·03 (0·54; 1·97)	OR	NS
			CBT vs FS (network)	-	>12 months			0·6 (0·13; 2·69)	OR	NS
			CBT vs RG (network)	-	>12 months			0·89 (0·25; 3·21)	OR	NS
			CBT vs CM (network)	-	>12 months			0·83 (0·34; 2·01)	OR	NS
			CBT vs PE (network)	-	>12 months			0·79 (0·35; 1·77)	OR	NS
			CBT vs TI (network)	-	>12 months			0·8 (0·2; 3·24)	OR	NS
			CBT vs ACT (network)	-	>12 months			0·63 (0·22; 1·83)	OR	NS
			CBT vs RE (network)	-	>12 months			0·5 (0·21; 1·17)	OR	NS
			CBT vs II (network)	-	>12 months			0·48 (0·21; 1·1)	OR	NS
			CBT vs CT (network)	-	>12 months			0·33 (0·08; 1·43)	OR	NS
			CBT vs HE (network)	-	>12 months			0·21 (0·03; 1·66)	OR	NS
			CBT vs SST (network)	-	>12 months			0·78 (0·34; 1·79)	OR	NS
			CBT vs TAU (pairways)	3	<6 months	-		0·67 (0·28; 1·57)	OR	NS
			CBT vs ST (network)	-	>12 months			0·96 (0·41; 2·26)	OR	NS
	Pilling et al. 2002		CBT vs active treatment	4	EoT	238		0·74 (0·42; 1·28)	OR	NS
			CBT vs other treatments	6	EoT	363		0·69 (0·44; 1·1)	OR	NS
			CBT vs standard care	3	EoT	163		0·73 (0·36-1·47)	OR	NS
			CBT vs all treatments	4	9-18 months	161		0·83 (0·16-4·24)	OR	NS
Social functioning	-									

Functioning and distress	Bighelli et al. 2018		CBT vs TAU (pairways)	9	EoT	734		-0·25 (-0·48; -0·03)	SMD	sig.
	Bighelli et al. 2021		CBT vs TAU (pairways)	5	EoT	-		-0·49 (-0·93; -0·06)	OR	sig.
			CBT vs TAU (network)	-	EoT	-		-0·25 (-0·48; -0·03)	SMD	sig.
	Wykes et al. 2008		CBT vs TAU	15	EoT	867		0·378 (0·154-0·602)	SMD	sig.
	Lincoln et al. 2008		CBT vs TAU	4	-	83		0·82 (0·07; 1·71)	SMD	-
	Bighelli et al. 2021		CBT vs TAU (network)	-	EoT			-0·63 (-1·06; -0·2)	SMD	NS
			CBT vs FI (network)	-	EoT			-0·80 (-1·54; -0·05)	SMD	NS
			CBT vs MIF (network)	-	EoT			-0·63 (-1·74; 0·48)	SMD	NS
			CBT vs SST (network)	-	EoT			-0·32 (-1·72; 1·07)	SMD	NS
			CBT vs PE (network)	-	EoT			0·05 (-1·05; 1·15)	SMD	NS
			CBT vs RG (network)	-	EoT			-0·06 (-0·84; 0·72)	SMD	NS
			CBT vs MI (network)	-	EoT			-0·05 (-1·11; 1·01)	SMD	NS
			CBT vs II (network)	-	EoT			-0·12 (-0·85; 0·62)	SMD	NS
			CBT vs ST (network)	-	EoT			-0·2 (-1·13; 0·74)	SMD	NS
			CBT vs FS (network)	-	EoT			-0·25 (-1·5; 0·99)	SMD	NS
			CBT vs ACT (network)	-	EoT			-0·27 (-0·99; 0·46)	SMD	NS
			CBT vs FP (network)	-	EoT			-0·32 (-0·94; 0·3)	SMD	NS
			CBT vs CM (network)	-	EoT			-0·64 (-1·42; 0·14)	SMD	NS
	Bighelli et al. 2018		CBT vs HFIT (network)	-	EoT	-		-0·33 (-1·05; 0·39)	SMD	NS
			CBT vs MF (network)	-	EoT	-		-0·29 (-1·44; 0·85)	SMD	NS
			CBT vs ST (network)	-	EoT	-		-0·13 (-0·83; 0·57)	SMD	NS
			CBT vs FI (network)	-	EoT	-		-0·23 (-0·87; 0·42)	SMD	NS
			CBT vs WL (network)	-	EoT	-		-0·24 (-0·70; 0·22)	SMD	NS
			CBT vs ACT (network)	-	EoT	-		-0·39 (-1·09; 0·32)	SMD	NS
			CBT vs IC (network)	-	EoT	-		-0·34 (-0·70; 0·02)	SMD	NS
			CBT vs IC (pairways)	3	EoT	315		-0·34 (-0·70; 0·02)	SMD	NS
Quality of life	Bighelli et al. 2018		CBT vs TAU (pairways)	5	EoT	401			SMD	sig.
			CBT vs TAU (network)	-	EoT	-			SMD	sig.
	Naeem et al 2016		B-CBT vs TAU	3	EoT				Hedges' G	<0.005
	Bighelli et al. 2018		CBT vs HFIT (network)	-	EoT	-			SMD	NS
			CBT vs WL (network)	-	EoT	-			SMD	NS
Suicide	Bighelli et al. 2021		CBT vs CM (network)	-	EoT			0·39 (0·01; 17·39)	OR	NS
			CBT vs FP (network)	-	EoT			0·62 (0·01; 35·28)	OR	NS
			CBT vs FS (network)	-	EoT			0·41 (0; 76·56)	OR	NS
			CBT vs PE (network)	-	EoT			0·12 (0; 13·77)	OR	NS
			CBT vs RG (network)	-	EoT			0·82 (0·02; 42·16)	OR	NS
			CBT vs FI (network)	-	EoT			0·56 (0·01; 21·19)	OR	NS
			CBT vs ACT (network)	-	EoT			0·54 (0·01; 39·26)	OR	NS
			CBT vs II (network)	-	EoT			0·52 (0·01; 41·27)	OR	NS
			CBT vs ST (network)	-	EoT			0,96 (0,06; 15,56)	OR	NS
			CBT vs TAU (network)	-	EoT			0,60 (0,02; 22,76)	OR	NS

Abbreviations: CBT: cognitive behavioral therapy, TAU: treatment as usual, end of treatment, NMA: network meta-analysis, PWA: pairwise meta-analysis, ES: effect size, MD: mean difference, OR: odds ratio, SMD: standard mean difference, NS: non-significant, CI: confidence interval, TAU1: , TAU2: , MPE: Mindfulness-based

psychoeducation, PE: Psycho-education, BF: befriending, FP: family psychoeducation, MIF: , WL: waiting list, B-CBT: , OPT: Other psychological treatments, PANSS: Positive and Negative Syndrome Scale, BPRS: Brief Psychiatric Rating Scale, CPS: , AT: active treatment, MCT: Metacognitive therapy, SST: Social skills training, CRSS: Cognitive remediation focused on social cognition, EMDR: Eye movement desensitization and reprocessing, FT: family therapy, HIT: Hallucinations focused integrative therapy, ALL: Protocol with 4 psychotherapies combined, CR: Cognitive remediation; CR_MCT: Cognitive remediation with Metacognitive therapy, WB: Wellbeing, Occupational therapy, SC: Supportive counselling, CT: Cognitive training, II: Integrated intervention, TI: Telemedicine, HE: health education, ACT: Acceptance and commitment therapy, HFIT: Hallucination focused integrative treatment, IC: Inactive control, MT: Metacognitive training, CM: Case management, RG: Relatives groups, EFC: Experience focused counselling, MF : Mindfulness, FS: Family support.

Supplemental Table 4. Sensitivity analysis, with only comparisons that include > 20 RCTs.

Outcome	Authors and year		Intervention and control group	RCTs	Follow-up	Sample size		Hedges' G*	P-value	I² (%)	PI 95CI%	SSE/ESB/LS	CE
General psychopathology	Jauhar et al. 2014		CBT vs TAU (68%), Unknown (19%), BF (3%), PE (3%), SC (2%), SAT (2%), CR (1%), ST(1%), GS (1%)	34	EoT	2991		-0·33 (-0·47; -0·19)	0·42 x10^{-5}	67·93	Notnull	No/No/Yes	III
Positive symptoms	Jauhar et al. 2014		TAU (60%), Unknown (21%), PE (5%), GF-SC (3%), BF (2%), SAT (2%), SC (2%), RS (2%), CR (2%), ST (1%), GS (0%)	33	EoT	2452		-0·26 (-0·37 ; -0·14)	0·20 x 10^{-4}	52·44	Null	No/No/Yes	III
Negative symptoms	Jauhar et al. 2014		TAU (59%), CR (10%), Unknown** (8%), BF (6%), PE (5%), GFT (3%), SAT (2%), SC (2%), RS (2%), ST (2%), GS (1%)	34	EoT	2354		-0·13 (-0·24; -0·02)	0·02	51·28	Null	No/No/Yes	IV
	Velthorst et al. 2015		CBT vs TAU (60%), BF (9%), PE (6%), unknown (5%), ETAU (4%), WL (3%), GFSC (3%), E-ST (3%), WL+TAU (3%), SC (2%), ST (2%)	28	EoT	2067		0·12 (-0·03; 0·27)	0·13	64·63	Null	Yes/No/No	NS
Delusions	Turner, Burger et al. 2020		CBT vs TAU (76%, SC (20%), PE (3%), ET (1%)	27	EoT	2169		0·36 (0·22; 0·51)	0·15 x10^{-5}	50·47	Null	Yes/No/No	III
Hallucinations	Turner, Burger et al. 2021		CBT vs TAU (72%), SC (24%), PE (3%), WL (1%)	28	EoT	2388		0·32 (0·19; 0·46)	0·41 x10^{-5}	45·14	Null	No/No/Yes	III
Functioning and distress	Bighelli et al. 2023		CBT vs TAU (64%), inactive control (8%), ST (10%), CR (9%), WL (6%), FI (1%), psychodynamic therapy (1%), PE (1%)	32	EoT	1328		0·24 (0·11; 0·38)	0·50 e-03	58·18	Null	No/No.No	III
Functioning and distress	Bighelli et al. 2023		CBT vs TAU (64%), inactive control (8%), ST (10%), CR (9%), WL (6%), FI (1%), psychodynamic therapy (1%), PE (1%)	30	EoT	2657		0·24 (0·11; 0·38)	0·50x10^{-3}	58·18	Null	No/No.No	III

			CBT vs TAU (61%), CR (14%), ST (9%), medication (5%), SAT (5%), BF (4%), PE (2%), WL (1%)	26	EoT	1704		0·25 (0·10; 0·40)	0·76 x10^{-3}	54·50	Null	No/No/No	IV

Abbreviations: PI: prediction interval, SSE: small study effect, ESB: excess significance bias, LS: largest study, CE: class of evidence, RCT: randomized controlled trial, EoT: end of therapy, ES: effect size, CBT: cognitive behavioral therapy, TAU: treatment as usual, PE: psychoeducation, BF: befriending, SC: supportive counseling, SAT: Social Activity Therapy, CR: Cognitive remediation, ST: supportive therapy, GS: group support, GF-SC: Goal-focused supportive contact, RS: Recreation and Support, GFT: goal focused therapy, ETAU: enriched treatment as usual, WL: waiting list, E-ST: enhanced supportive therapy, ET: exposure therapy, FI: family intervention.

*in some cases the summary estimates from our analysis differed one-hundredth decimal compared to the original findings. We used the data provided by the report of the meta-analysis and expect that variation between estimates adopted from the primary RCTs and estimates provided in the meta-analysis have caused this slight and clinically irrelevant variation

Supplemental Table 5. The number of times that one randomized trial is included in a meta-analysis

Author	Year	Follow-up	Times included in a meta-analysis
Valmaggia et al.	2005	EoT	16
Durham et al.	2003	EoT	14
Haddock et al.	2009	EoT	13
Cather et al.	2005	EoT	11
Lewis et al.	2002	EoT	11
Bechdolf et al.	2004	EoT	10
Garety et al.	2008	Long	10
Pinto et al.	1999	EoT	10
Startup et al.	2004	EoT	10
Krakvik et al.	2013	EoT	9
Lincoln et al.	2012	EoT	9
Morrison et al.	2014	EoT	9
Barrowclough et al.	2006	EoT	8
Garety,Carer et al.	2008	EoT	8
Morrison et al.	2018	EoT	8
Farhall et al.	2009	EoT	7
Penn et al.	2009	Long	7
Garety et al.	2008	EoT	6
Gaudiano et al.	2006	EoT	6
Granholm et al.	2005	EoT	6
Gumley et al.	2003	EoT	6
Haddock et al.	2009	Long	6
Penades et al.	2006	EoT	6
Penn et al.	2009	EoT	6
Penn et al.	2009	Medium	6
Rathod et al.	2013	EoT	6
Rector et al.	2003	EoT	6
Sensky et al.	2000	EoT	6
Valmaggia et al.	2005	Medium	6
Bechdolf et al.	2004	Long	5
Bechdolf et al.	2004	Medium	5
Drury et al.	1996	EoT	5
Garety,No,Carer et al.	2008	EoT	5
Haddock et al.	2009	Short	5
Jackson et al.	2008	EoT	5
Lewis et al.	2002	Long	5
Tarrier et al.	2014	EoT	5
Vandergaag et al.	2011	EoT	5
Wykes et al.	2005	EoT	5
Barrowclough et al.	2001	Medium	4

Barrowclough et al.	2014	Long	4
Durham et al.	2003	Long	4
Edwards_Clz et al.	2011	EoT	4
Edwards_Tdz et al.	2011	EoT	4
Foster et al.	2010	EoT	4
Gottlieb et al.	2017	EoT	4
Klingberg et al.	2011	EoT	4
Lecomte et al.	2008	EoT	4
Levine et al.	1998	EoT	4
Lewis et al.	2002	Short	4
Lewis_L et al.	2002	Long	4
Lewis_M et al.	2002	Long	4
Lewis_N et al.	2002	Long	4
Li et al.	2014	Long	4
Li et al.	2014	Medium	4
Li et al.	2015	Short	4
Penn et al.	2009	Short	4
Peters et al.	2010	EoT	4
Startup et al.	2004	Medium	4
Tarrier et al.	1999	EoT	4
Tarrier et al.	1999	Long	4
Trower et al.	2004	EoT	4
Valmaggia et al.	2005	Short	4
Barretto et al.	2009	EoT	3
Barrowclough et al.	2001	Long	3
Barrowclough et al.	2010	Long	3
Bechdolf et al.	2004	Short	3
Birchwood et al.	2014	EoT	3
Bradshaw et al.	2000	EoT	3
Farhall et al.	2009	Long	3
Fowler et al.	2009	EoT	3
Fowler et al.	2009	Long	3
Freeman et al.	2014	EoT	3
Freeman et al.	2015	EoT	3
Granholm et al.	2002	EoT	3
Granholm et al.	2014	EoT	3
Gumley et al.	2003	Long	3
Guo et al.	2015	Long	3
Haddock et al.	1999	EoT	3
Hall et al.	2003	EoT	3
Hall-Tarier et al.	2003	EoT	3
Jackson et al.	2008	Long	3
Jolley et al.	2003	EoT	3
Leclerc et al.	2000	EoT	3

Li et al.	2014	Short	3
Li et al.	2015	EoT	3
Mcleod et al.	2007	EoT	3
Naeem et al.	2016	EoT	3
Naeem et al.	2016	EoT	3
Pinninti et al.	2010	EoT	3
Rathod et al.	2012	EoT	3
Shawyer et al.	2012	EoT	3
Tarrier et al.	2014	Medium	3
Waller et al.	2015	EoT	3
Wang et al.	2003	EoT	3
Barretto et al.	2009	Medium	2
Barrowclough et al.	2006	Medium	2
Barrowclough et al.	2010	Medium	2
Cather et al.	2005	Short	2
Daniels et al.	1998	EoT	2
Drury et al.	2000	Long	2
Durham et al.	2003	Medium	2
Gleeson et al.	2009	Medium	2
Granholm et al.	2005	Long	2
Granholm et al.	2005	Medium	2
Granholm et al.	2012	EoT	2
Granholm et al.	2013	EoT	2
Granholm et al.	2013	Long	2
Grawe et al.	2006	Long	2
Habib et al.	2015	EoT	2
Husain et al.	2017	EoT	2
Kingsep et al.	2003	EoT	2
Kuipers et al.	1997	EoT	2
Leff et al.	2013	EoT	2
Levine et al.	1998	Short	2
Morrison et al.	2018	Medium	2
Naeem et al.	2015	EoT	2
Odonnel et al.	2003	Long	2
Pan et al.	2012	Short	2
Qiu et al.	2014	Medium	2
Sensky et al.	2000	Long	2
Startup et al.	2004	Long	2
Steel et al.	2017	EoT	2
Tarrier et al.	1998	EoT	2
Valmaggia et al.	2005	FollowUp	2
Wang et al.	2015	Long	2
White et al.	2011	EoT	2
Wong et al.	2019	EoT	2

Zhang et al.	1999	EoT	2
Baker et al.	2006	EoT	1
Baker et al.	2006	Long	1
Baker et al.	2006	Medium	1
Barretto et al.	2009	FollowUp	1
Barrowclough et al.	2001	EoT	1
Barrowclough et al.	2006	Long	1
Barrowclough et al.	2014	Medium	1
Bechdolf et al.	2010	EoT	1
Birchwood et al.	2014	Long	1
Birchwood et al.	2014	Medium	1
Buchkremer et al.	1997	Long	1
Buchkremer et al.	1997	Medium	1
Cao et al.	2014	Long	1
Chen et al.	2015	Medium	1
Chen et al.	2015	Short	1
Craig et al.	2017	EoT	1
Deng et al.	2008	EoT	1
Durham et al.	2003	Short	1
Durham_A et al.	2003	EoT	1
Durham_B et al.	2003	EoT	1
Edwards et al.	2011	Short	1
Edwards_Clz et al.	2011	Long	1
Edwards_Tdz et al.	2011	Long	1
England et al.	2007	Long	1
England et al.	2007	Short	1
England et al.	2008	EoT	1
Foster et al.	2010	Medium	1
Freeman et al.	2014	Medium	1
Freeman et al.	2015	Medium	1
Freeman et al.	2015	Short	1
Freeman et al.	2016	EoT	1
Freeman_A et al.	2015	EoT	1
Freeman_B et al.	2015	EoT	1
Freeman_C et al.	2014	EoT	1
Freeman_S et al.	2014	EoT	1
Garety et al.	2008	Medium	1
Garety et al.	2008	Short	1
Granholm et al.	2013	Medium	1
Granholm et al.	2013	Short	1
Granholm et al.	2014	Long	1
Granholm et al.	2014	Medium	1
Granholm et al.	2014	Short	1
Granholm et al.	2020	EoT	1

Grant et al.	2011	EoT	1
Grant et al.	2012	EoT	1
Guo et al.	2015	Medium	1
Guo et al.	2015	Short	1
Haddock et al.	1999	Long	1
Haddock et al.	1999	Short	1
Hall et al.	2003	Long	1
Hall et al.	2003	Medium	1
Halperin et al.	2000	EoT	1
Hayward et al.	2017	EoT	1
Hazell et al.	2017	EoT	1
Hjorthoj et al.	2013	EoT	1
Hogarty et al.	1997	Long	1
Hu et al.	2013	Medium	1
Jackson et al.	2007	EoT	1
Jia et al.	2005	Short	1
Jiao et al.	2014	Short	1
Kemp et al.	1998	Long	1
Kemp et al.	1998	Short	1
Klingberg et al.	2012	Long	1
Kuipers et al.	1997	Long	1
Kuipers et al.	1997	Medium	1
Lecomte et al.	2008	Medium	1
Lecomte et al.	2008	Short	1
Lecomte_A et al.	2008	EoT	1
Lecomte_B et al.	2008	EoT	1
Lee et al.	2012	EoT	1
Lee et al.	2013	EoT	1
Lewis et al.	2002	Medium	1
Li et al.	2013	Short	1
Li et al.	2014	EoT	1
Ma et al.	2016	Short	1
Madigan et al.	2013	EoT	1
Madigan et al.	2013	Long	1
Morrison et al.	2014	Long	1
Morrison et al.	2014	Medium	1
Morrison et al.	2018	FollowUp	1
Morrison et al.	2018	Long	1
Pan et al.	2012	Long	1
Pan et al.	2012	Medium	1
Panninti et al.	2010	EoT	1
Panninti et al.	2010	Medium	1
Penades et al.	2006	Long	1
Penn et al.	2015	EoT	1

Peters et al.	2010	Medium	1
Pos et al.	2019	EoT	1
Pot-Kolder et al.	2016	EoT	1
Potkolder et al.	2018	EoT	1
Qjan et al.	2012	Long	1
Qiu et al.	2014	Short	1
Rathod et al.	2013	Medium	1
Rector et al.	2003	Medium	1
Shawyer et al.	2012	Medium	1
Startup et al.	2005	EoT	1
Steel et al.	2017	Long	1
Sun et al.	2014	Short	1
Tarrier et al.	1993	EoT	1
Tarrier et al.	1999	Short	1
Tarrier et al.	2004	EoT	1
Tarrier et al.	2004	Long	1
Tarrier et al.	2014	Long	1
Tarrier_A et al.	1998	EoT	1
Tarrier_B et al.	1998	EoT	1
Turkington et al.	2000	EoT	1
Turkington et al.	2002	EoT	1
Turkington et al.	2002	Medium	1
Turkington et al.	2006	Medium	1
Valmaggia et al.	2005	Long	1
Valmaggia et al.	2006	EoT	1
Velligan et al.	2014	EoT	1
Waller et al.	2015	Medium	1
Waller et al.	2018	EoT	1
Wang et al.	2005	Short	1
Wang et al.	2008	Short	1
Wang et al.	2015	Medium	1
Wang et al.	2015	Short	1
Wu et al.	2008	EoT	1
Zhao et al.	2013	Short	1
Zhao et al.	2014	Short	1

Supplemental figures 1-34. Forest plots for each outcome per follow-up assessment.

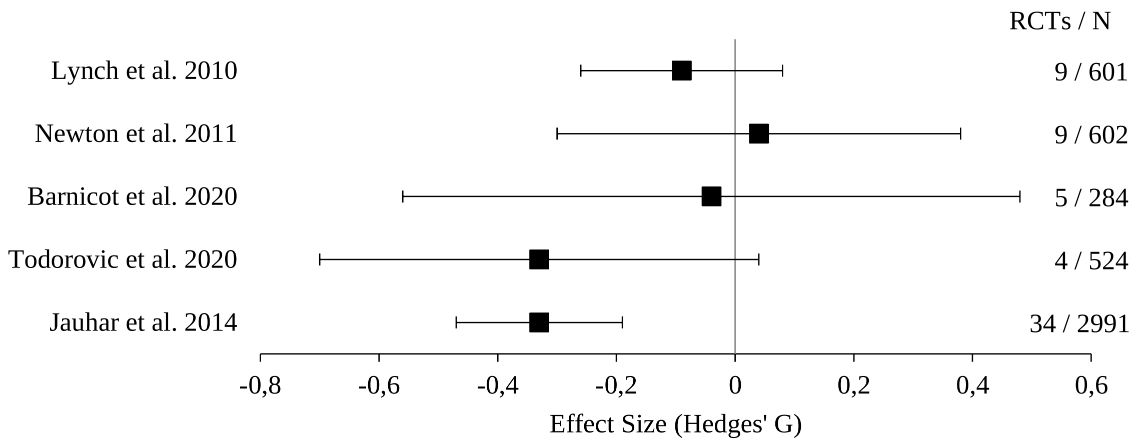

Figure 1. General psychopathology, end of therapy, randomized controlled trials (RCTs) and total sample size (N).

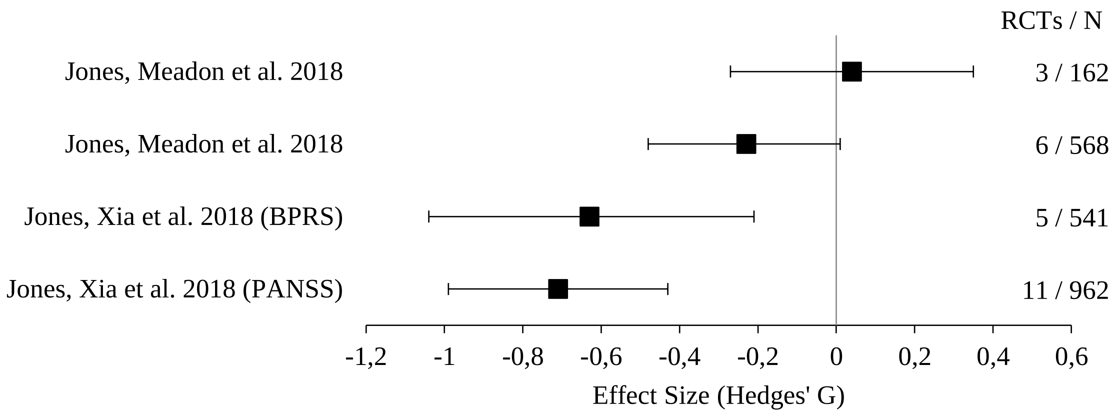

Figure 2. General psychopathology, follow-up of 0-6 months, randomized controlled trials (RCTs) and total sample size (N).

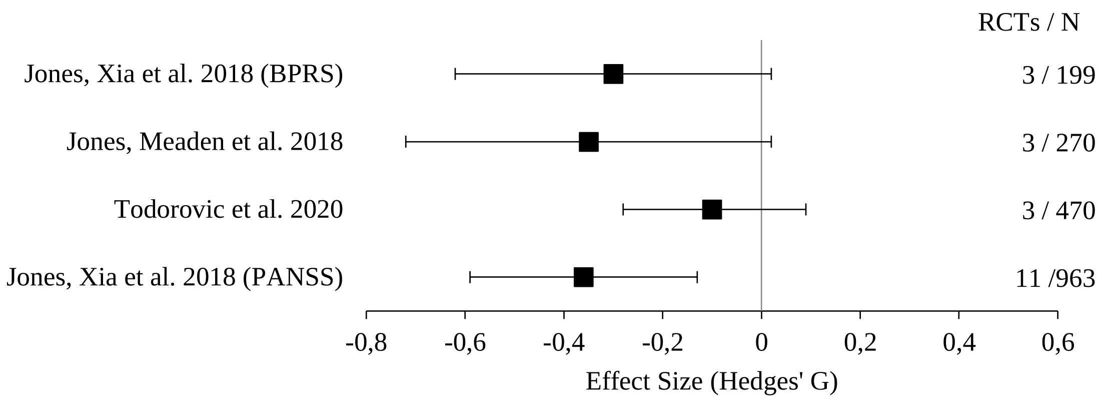

Figure 3. General psychopathology, follow-up 6-12 months, randomized controlled trials (RCTs) and total sample size (N).

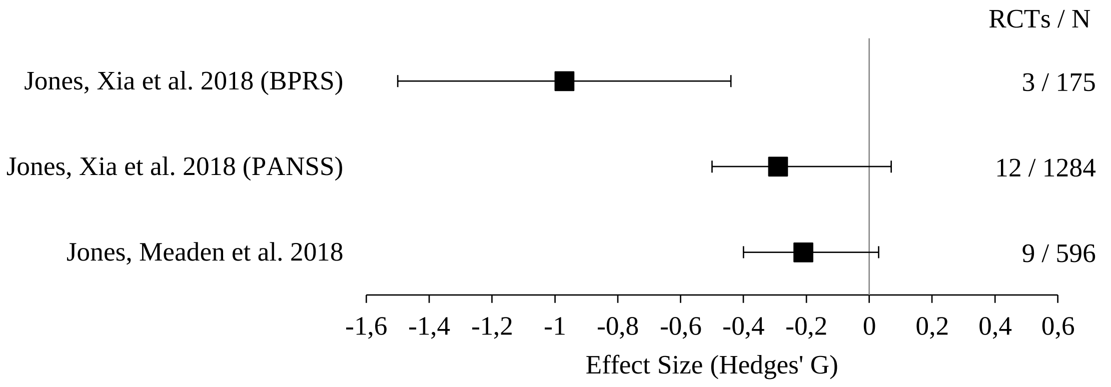

Figure 4. General psychopathology, follow-up more than 12 months, randomized controlled trials (RCTs) and total sample size (N).

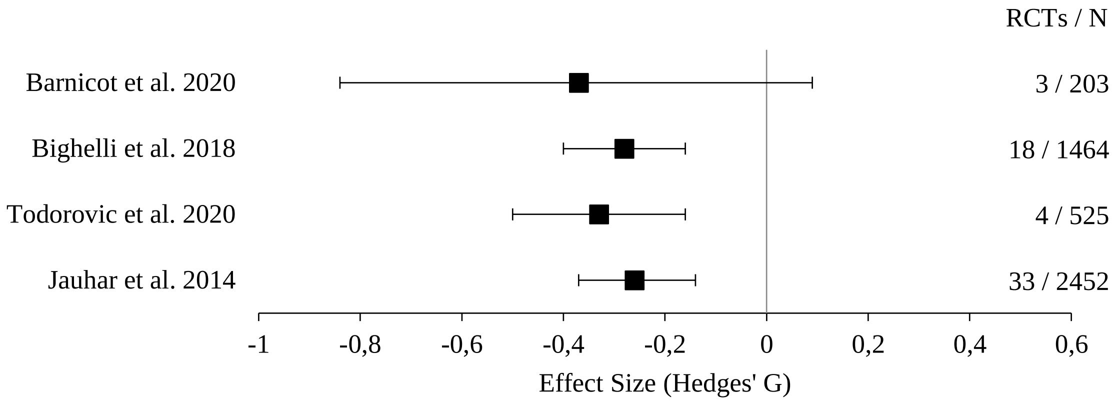

Figure 5. Positive symptoms, end of therapy, randomized controlled trials (RCTs) and total sample size (N).

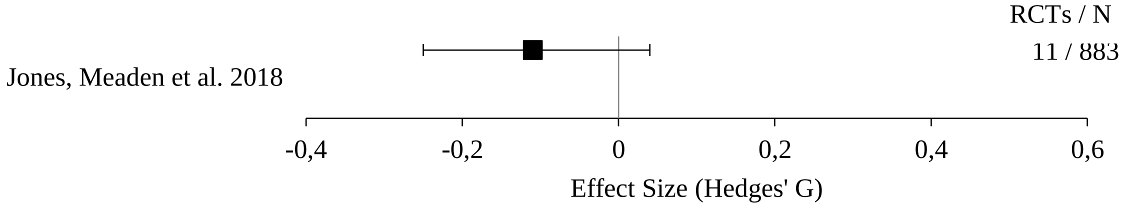

Figure 6. Positive symptoms, follow-up 0-6 months, randomized controlled trials (RCTs) and total sample size (N).

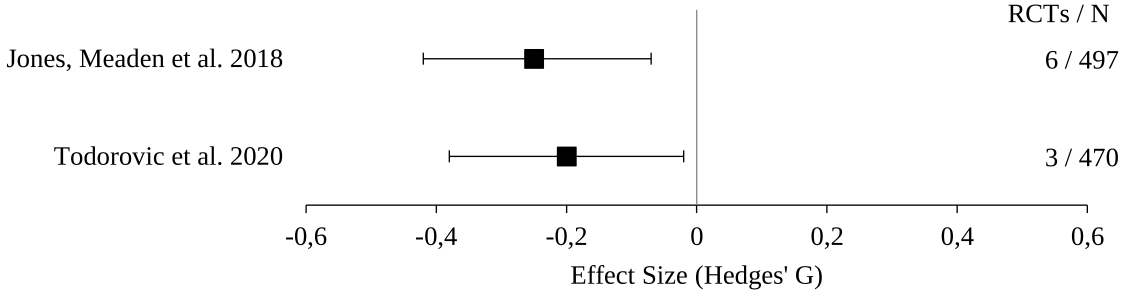

Figure 7. Positive symptoms, follow-up 6-12 months, randomized controlled trials (RCTs) and total sample size (N).

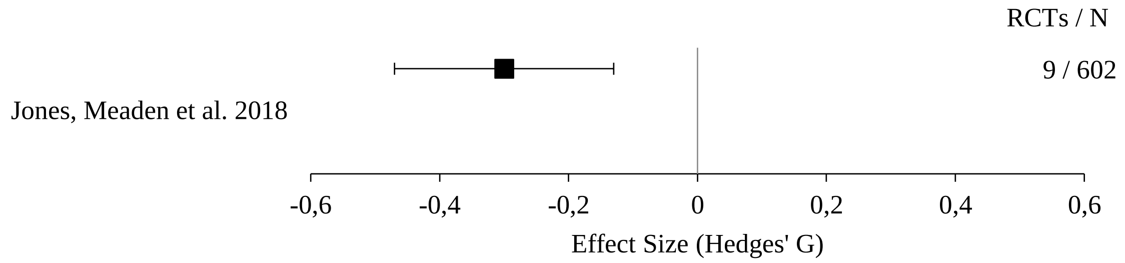

Figure 8. Positive symptoms, follow-up more than 12 months, randomized controlled trials (RCTs) and total sample size (N).

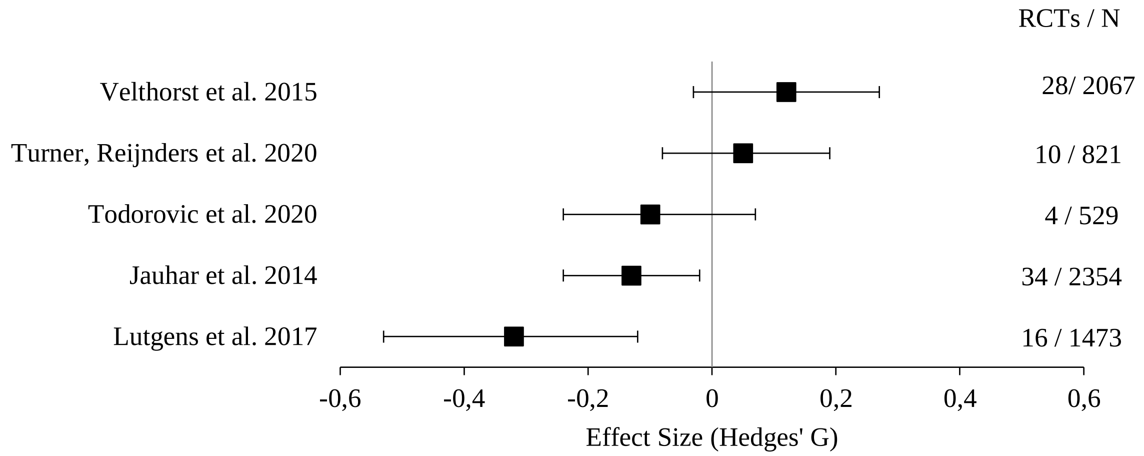

Figure 9. Negative symptoms, end of therapy, randomized controlled trials (RCTs) and total sample size (N).

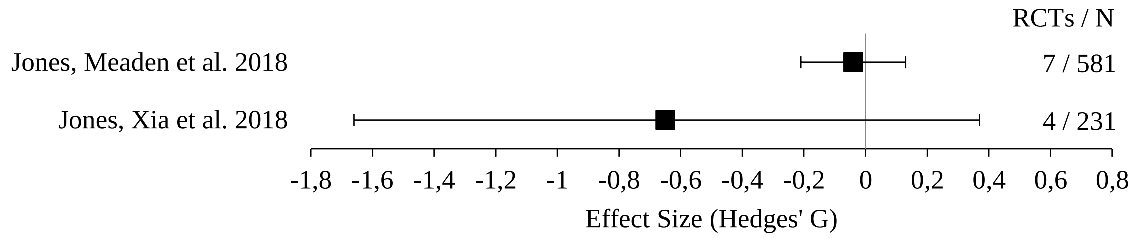

Figure 10. Negative symptoms, follow-up of 0-6 months, randomized controlled trials (RCTs) and total sample size (N).

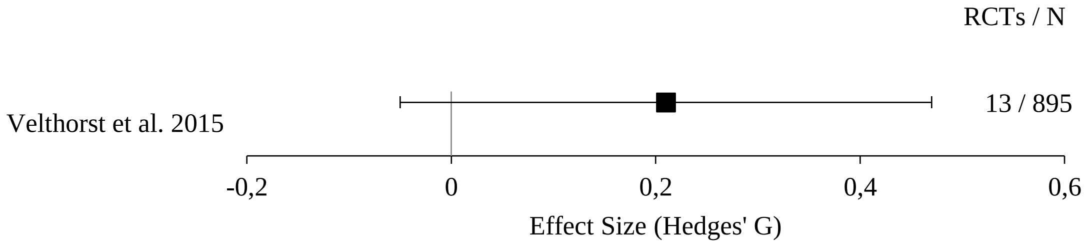

Figure 11. Negative symptoms, follow-up of 3-6 months, randomized controlled trials (RCTs) and total sample size (N).

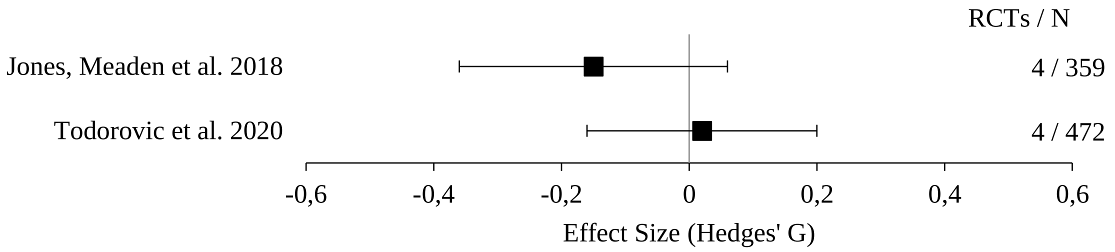

Figure 12. Negative symptoms, follow-up of 6-12 months, randomized controlled trials (RCTs) and total sample size (N).

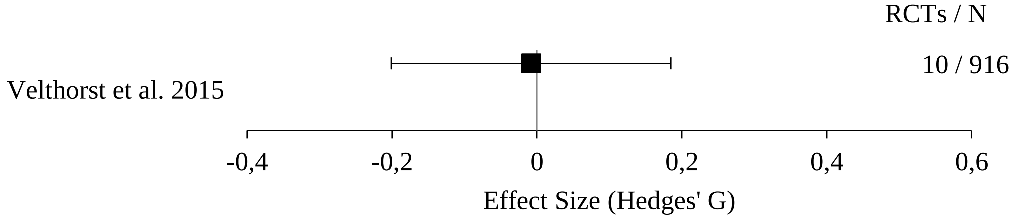

Figure 13. Negative symptoms, follow-up of 9-12 months, randomized controlled trials (RCTs) and total sample size (N).

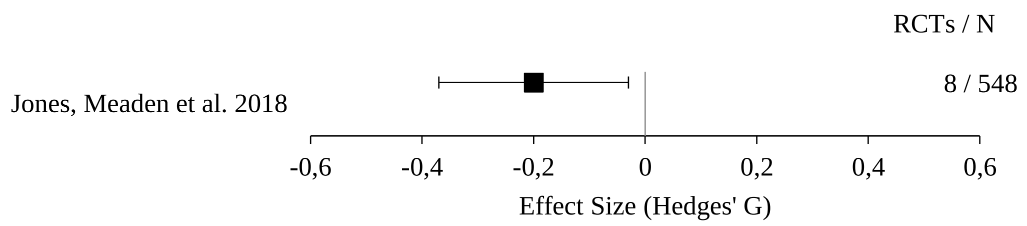

Figure 14. Negative symptoms, follow-up of more than 12 months, randomized controlled trials (RCTs) and total sample size (N).

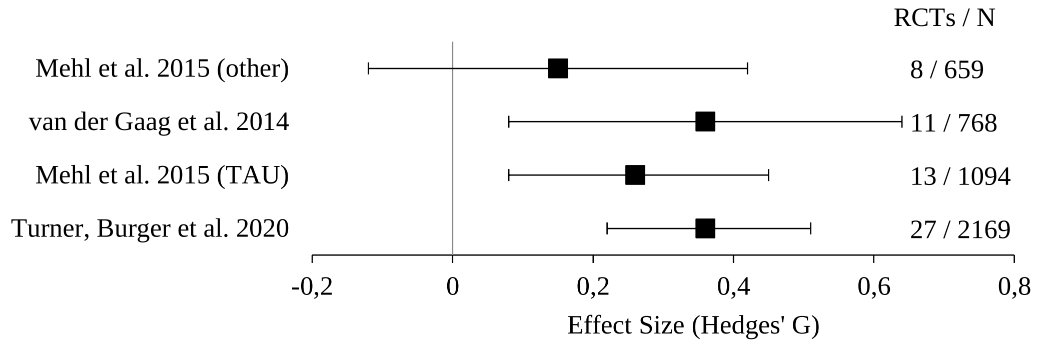

Figure 15. Delusions, end of therapy, randomized controlled trials (RCTs) and total sample size (N).

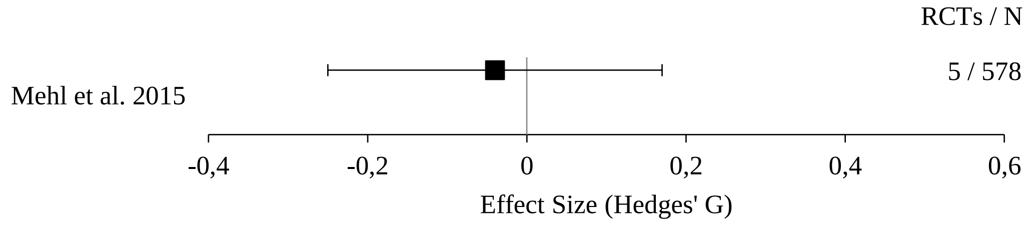

Figure 16. Delusions, follow-up less than 9 months, randomized controlled trials (RCTs) and total sample size (N). .

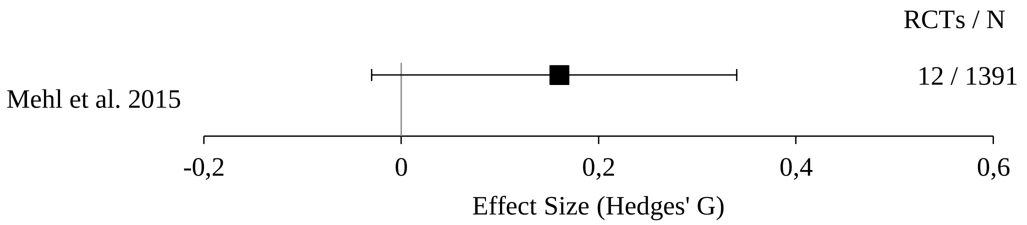

Figure 17. Delusions, follow-up more than 12 months, randomized controlled trials (RCTs) and total sample size (N).

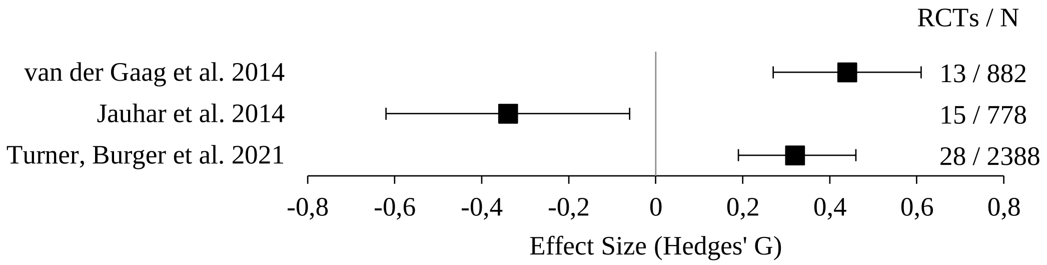

Figure 18. Hallucinations, end of therapy, randomized controlled trials (RCTs) and total sample size (N).

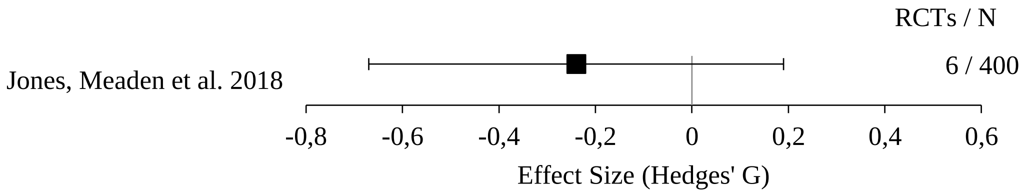

Figure 19. Affective symptoms, follow-up of 0-6 months, randomized controlled trials (RCTs) and total sample size (N).

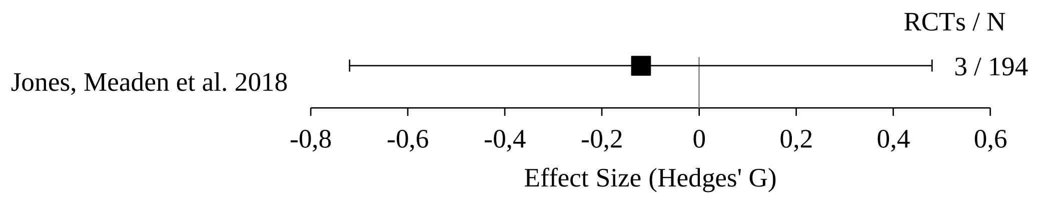

Figure 21. Affective symptoms, follow-up of 6-12 months, randomized controlled trials (RCTs) and total sample size (N).

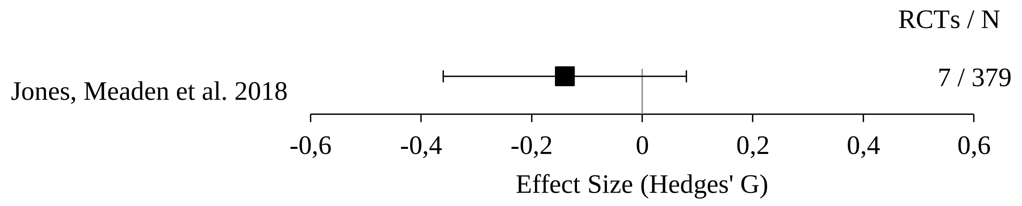

Figure 22. Affective symptoms, follow-up more than 12 months, randomized controlled trials (RCTs) and total sample size (N).

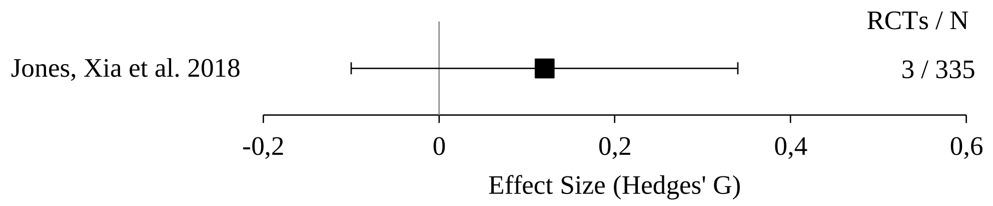

Figure 23. Anxiety, follow-up more than 12 months, randomized controlled trials (RCTs) and total sample size (N).

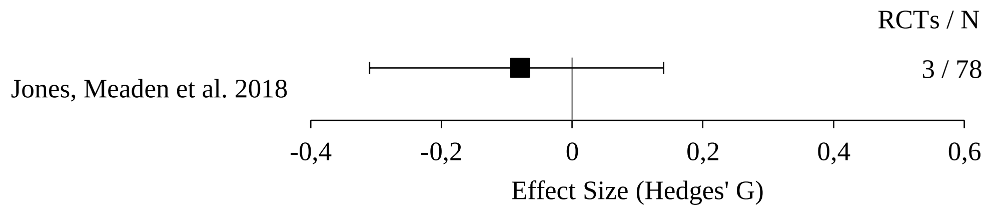

Figure 24. Rehospitalisation, follow-up of 6-12 months, randomized controlled trials (RCTs) and total sample size (N).

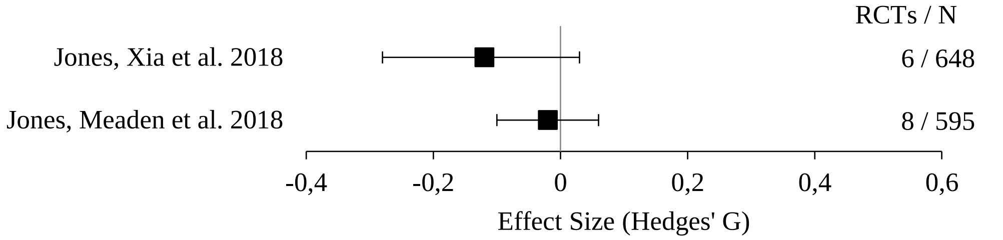

Figure 25. Rehospitalisation, follow-up of more than 12 months, randomized controlled trials (RCTs) and total sample size (N).

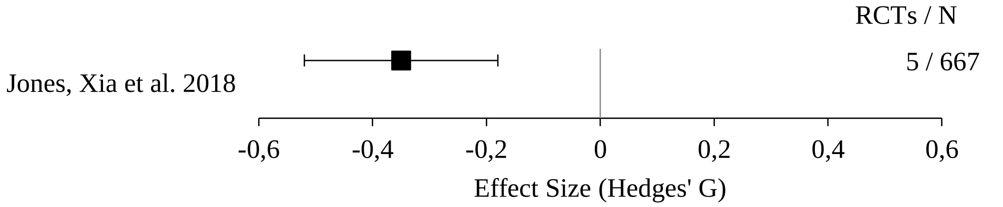

Figure 26. Relapse, follow-up of 6-12 months, randomized controlled trials (RCTs) and total sample size (N).

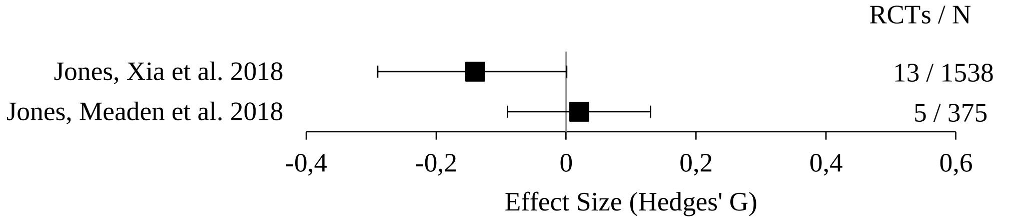

Figure 27. Relapse, follow-up of more than 12 months, randomized controlled trials (RCTs) and total sample size (N).

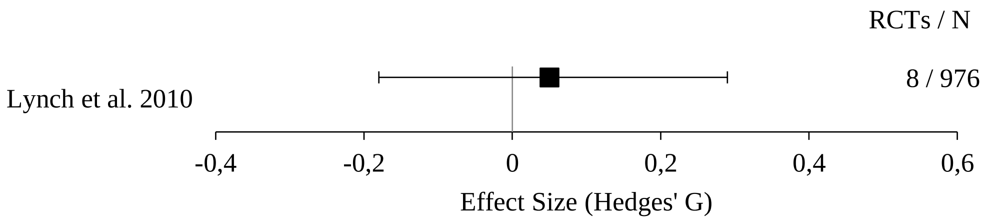

Figure 28. Relapse, follow-up of 6-36 months, randomized controlled trials (RCTs) and total sample size (N).

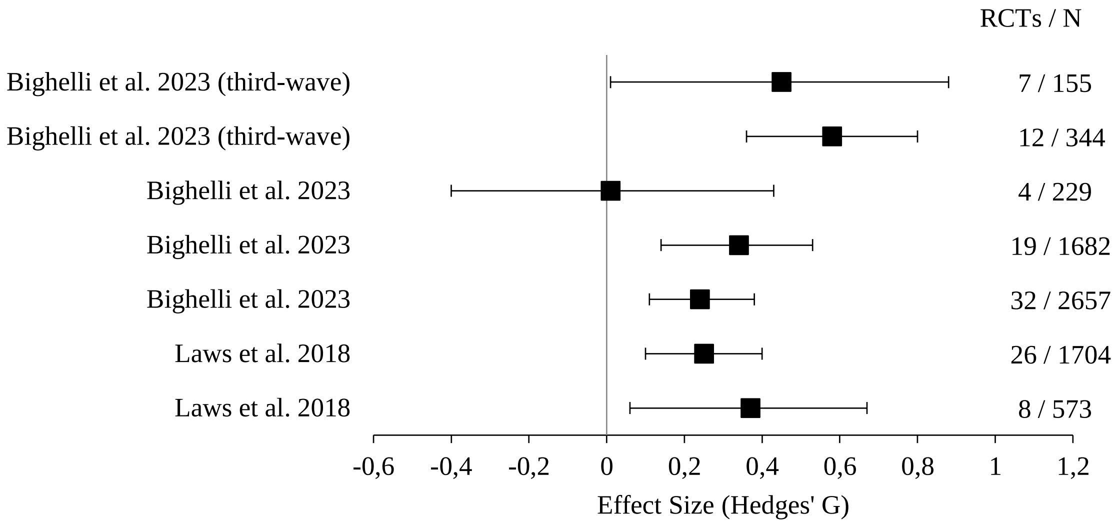

Figure 29. Functioning and distress, end of therapy, randomized controlled trials (RCTs) and total sample size (N).

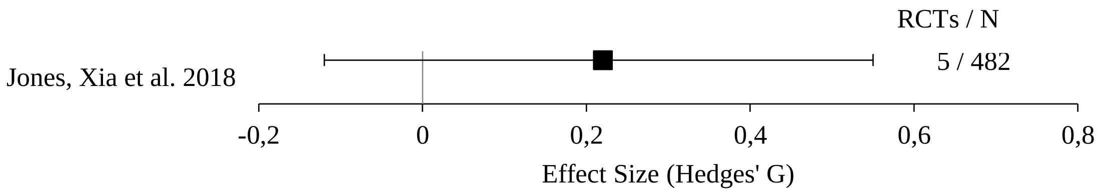

Figure 30. Functioning and distress, 0-6 months, randomized controlled trials (RCTs) and total sample size (N).

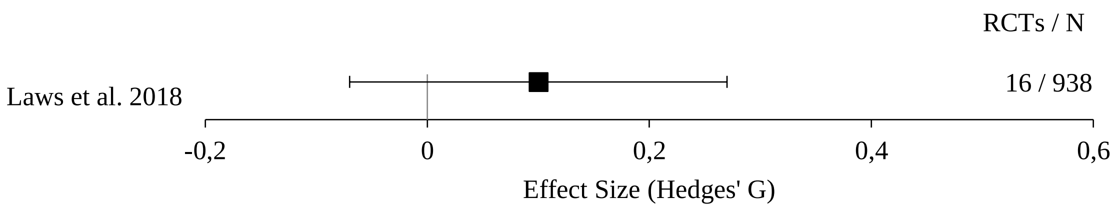

Figure 31. Functioning and distress, 3-18 months, randomized controlled trials (RCTs) and total sample size (N).

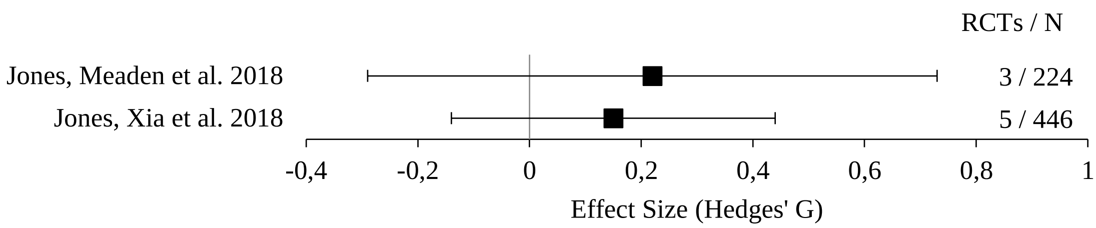

Figure 32. Functioning and distress, more than 12 months, randomized controlled trials (RCTs) and total sample size (N).

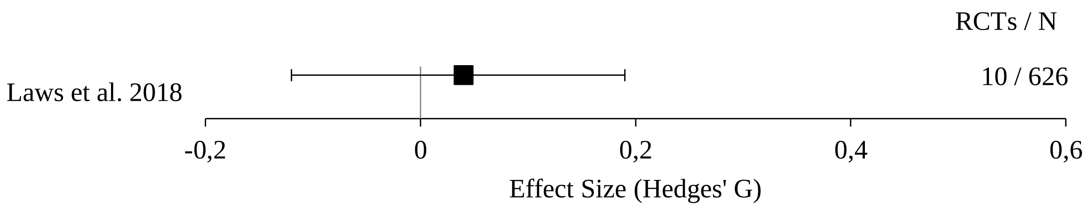

Figure 33. Quality of life, end of therapy, randomized controlled trials (RCTs) and total sample size (N).

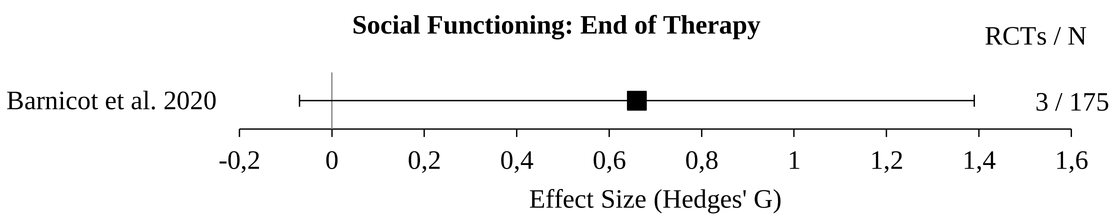

Figure 34. Social functioning, end of therapy, randomized controlled trials (RCTs) and total sample size (N).
